# Not all birds have a single dominantly expressed MHC-I gene: Transcription suggests that siskins have many highly expressed MHC-I genes

**DOI:** 10.1038/s41598-019-55800-9

**Published:** 2019-12-20

**Authors:** Anna Drews, Helena Westerdahl

**Affiliations:** Department of Biology, Lund University, Lund, Sweden

**Keywords:** Molecular evolution, Evolution, Ecology, Molecular ecology

## Abstract

Passerine birds belong to the most species rich bird order and are found in a wide range of habitats. The extremely polymorphic adaptive immune system of passerines, identified through their major histocompatibility complex class I genes (MHC-I), may explain some of this extreme radiation. Recent work has shown that passerines have higher numbers of MHC-I gene copies than other birds, but little is currently known about expression and function of these gene copies. Non-passerine birds have a single highly expressed MHC-I gene copy, a pattern that seems unlikely in passerines. We used high-throughput sequencing to study MHC-I alleles in siskins (*Spinus spinus*) and determined gene expression, phylogenetic relationships and sequence divergence. We verified between six and 16 MHC-I alleles per individual and 97% of these were expressed. Strikingly, up to five alleles per individual had high expression. Out of 88 alleles 18 were putatively non-classical with low sequence divergence and expression, and found in a single phylogenetic cluster. The remaining 70 alleles were classical, with high sequence divergence and variable degrees of expression. Our results contradict the suggestion that birds only have a single dominantly expressed MHC-I gene by demonstrating several highly expressed MHC-I gene copies in a passerine.

## Introduction

Gene duplication is an important evolutionary mechanism since it can result in a relaxed pressure for maintaining the original gene function^1,2^. There are several different ways for a gene to gain a new function, one is neo-functionalization where one of the newly duplicated gene copies can be free to evolve novel functions^1,2^. Another mechanism is sub-functionalization where the original function of the gene is split between the two gene copies^3^. One example of a gene region with a large number of duplicated genes is the Major Histocompatibility Complex (MHC)^4^. The MHC genes has been hypothesized to evolve through a mechanisms called the ‘birth and death process’ during which some gene copies retain their original function whereas others evolve slightly different functions and yet other gene copies become non-functional^5,6^. Some species are known to have a very large number of MHC gene copies, for example the Atlantic cod (*Gadus morhua*) and the sedge warbler (*Acrocephalus schoenobaenus*)^7,8^. With the use of high-throughput sequencing (HTS) it is now possible to accurately genotype MHC in species with highly duplicated MHC genes ^*e.g.*^^9–13^.

Classical MHC genes encode MHC molecules that have a central role in the vertebrate adaptive immune system where they present peptide antigens to T-cells to enable recognition and elimination of pathogens^4^. There are two classes of MHC molecules with central roles in adaptive immunity and they have slightly different functions: MHC class I (MHC-I) molecules present peptides derived from the cytoplasm to cytotoxic T-cells whereas MHC class II (MHC-II) molecules present peptides from the extra-cellular environment to T-helper cells^14^. The classical MHC genes are among the most polymorphic genes identified to date and this high polymorphism is maintained by balancing selection^4,15–18^. There is also a parallel set of MHC class I and II genes that are rather similar to the classical MHC genes, so called non-classical MHC genes. There are several different types of non-classical MHC genes but what they have in common is that they lack at least one of the defending features of classical MHC genes, *i*.*e*. high polymorphism, high gene expression and presentation of peptides to T-cells^19–21^. Non-classical genes are so far best described in mammals^19,22,23^. However, putatively non-classical genes have also been reported in birds from several different orders: Galliformes^24–27^, Anseriformes^28^, Pelecaniformes^29–31^, Charadriiformes^32^ and Passeriformes^33^.

Classical MHC-I genes have been thoroughly characterized in a handful of bird species that belong to the order Galliformes. The chicken (*Gallus gallus*), the turkey (*Meleagris gallopavo*) and the Japanese quail (*Coturnix japonica*) all have two classical MHC-I gene copies^34–38^. In chickens the two gene copies are expressed to different degrees; one has high gene expression – the major, and the other has low expression – the minor^39–41^. Recent research in two Galliform and one Anseriform species has found a single dominantly expressed classical MHC-I gene copy in the turkey, the Japanese quail and the mallard (*Anas platyrhynchos*)^37,42–45^. The combined evidence from these studies indicate that all birds have a single highly expressed classical MHC-I gene. Drews *et al*.^33^ recently corroborated this theory by demonstrating a single highly expressed MHC-I gene in two species of sparrows, the house sparrow (*Passer domesticus*) and the tree sparrow (*Passer montanus*), birds that belong to the order Passeriformes.

Passeriformes is the most species rich bird order on earth and species belonging to this order can be found worldwide and in a wide range of habitats. One reason for passerines ability to adapt to a wide range of different habitats could be that their MHC genes are highly duplicated and extremely polymorphic, hence passerines can potentially cope with a large diversity of pathogens. MHC-I and MHC-IIB genes have mostly been characterized on the gene level in passerine birds ^*e.g.*^^8,11,46,47^, and should therefore be referred to as MHC-I like and MHC-IIB like genes since their true function and genomic region have not been fully verified, though hereafter we of convenience call them just MHC-I and MHC-IIB genes. O’Connor *et al*.^48^ performed a comprehensive study of MHC-I using HTS across a large number of species from the parvorder Passerida within the order Passeriformes. These species had between seven and 37 MHC-I alleles, *i*.*e*. at least four to 19 MHC-I gene copies per individual (classical and non-classical alleles combined). The highest number of MHC-I gene copies reported in any passerine species to date is in the sedge warbler where 65 MHC-I alleles have been identified in a single individual^8^. In the zebra finch genome several MHC-I genes can be found but only one gene copy seem to be functional^49,50^ so the zebra finch seem to be an outlier among passerines. The exact purpose of this high number of MHC-I gene copies in passerines is not known and only limited efforts have been made to distinguish between classical and non-classical genes in passerines with even less research attention dedicated to studying MHC-I gene expression^33^.

In the present study, we therefore set out to investigate MHC-I gene expression in a passerine bird that is phylogenetically distant from sparrows to test whether a single highly expressed MHC-I gene copy is frequent also among passerines. We partly characterized MHC-I in the Eurasian siskin (*Spinus spinus*), and measured the relative expression of specific MHC-I alleles on the transcription level using HTS. We also wanted to investigate the possible existence of putatively non-classical alleles in siskins and these genes were defined based on three criteria: low relative gene expression (transcription), low sequence divergence and the feature of non-classical alleles to form a highly supported cluster in a phylogenetic tree. We then determined the degree of gene expression in putatively classical and non-classical MHC-I alleles. Finally, the phylogenetic relationships between the MHC-I alleles were evaluated in relation to their expression levels, sequence divergence and signs of positive selection.

## Results

### Characterization and genotyping of MHC-I in siskins

MHC-I in siskins was initially characterized by Sanger-sequencing exon 2 to 4 in a single siskin individual. The retrieved siskin alleles were easily aligned against other bird MHC-I sequences and sites known to be conserved across vertebrates were also present in the MHC-I of siskins (Supplementary Fig. 1). MHC-I exon 3 was subsequently amplified and sequenced using Illumina MiSeq in 18 individuals, including the individual where MHC-I was partly characterized. In order to retrieve the full MHC-I allelic divergence and the expressed MHC-I allele in each individual three different primer combinations were used, two primer combinations on gDNA (primer combination 1 and 2) and two on cDNA (RNA; primer combination 2 and 3). For each primer combination three or four samples were run twice, so called duplicates, and these duplicates were used in order to confirm that the results were stable and repeatable. All duplicates amplified the same alleles within each individual and had similar relative read depths (Supplementary Fig. 2). In total 88 different exon 3 alleles were verified and considered to be true alleles (GenBank acc. nr. MN686116-MN686203), eight out of the 88 alleles had a 3 bp insertion (Supplementary Fig. 3). 84 out of these 88 alleles were found in cDNA, *i*.*e*. they were expressed (transcribed). Siskins had 11 ± 3 ($\bar{{\rm{x}}}$ ± SD, reported from here and onwards) different gDNA alleles per individual, (minimum six alleles and maximum 16), and 11 ± 2 different cDNA alleles, (minimum six expressed alleles and maximum 14), *i*.*e*. most alleles were expressed.

### Expression and phylogenetic relationships among MHC-I alleles

The relative read depth per allele, a measure of degree of expression of specific MHC-I alleles, varied considerably between the 84 expressed alleles. An allele was considered to be highly expressed if it had a higher relative read depth than expected given that all alleles within an individual were expressed equally. Moreover, the classification (*i*.*e*. high or low expression) from primer combinations 2 and 3 also had to agree. Out of the 84 expressed alleles 59 were amplified with both primer combinations and most of the time the two primer combinations agreed on expression level (92%: 119 out of 130 cases where expression of alleles could be compared within individuals). In order to solve the cases where primer combinations 2 and 3 disagreed additional rules were applied (see Methods), resulting in a 98% match between the primer combinations. In the end we were able to place 80 of the 84 expressed alleles into one of the two groups, high or low expression, and these two groups were confirmed also using an independent test were the relative read depth in cDNA was adjusted for the relative read depth in gDNA (Supplementary Fig. 4).

**Figure 1:**
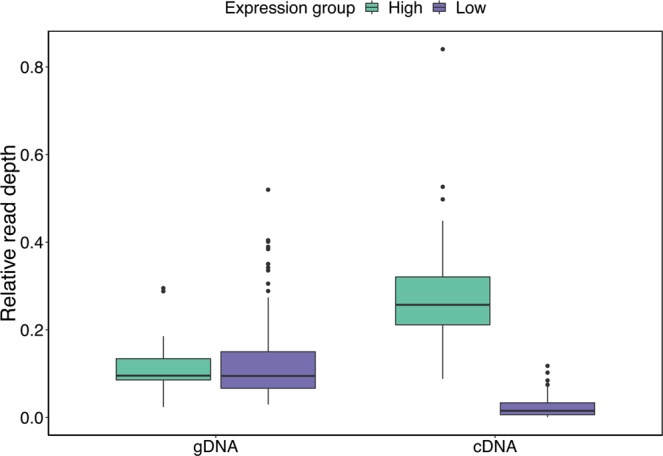
Boxplot of the relative read depth of every MHC-I allele in 18 siskin individuals for gDNA (genomic DNA) and cDNA (reverse transcribed mRNA). The data is presented separately for the high expression (green) and the low expression (purple) groups, and only alleles where the expression (relative read depth) could be defined are included. Note that this figure is only based on relative read depths from alleles amplified by primer combination 2 (N = 66 alleles in total) whereas the definition high and low expression was based on relative read depths from primer combination 2 and 3 combined (N = 76 alleles in total).

There was a significant discrepancy in relative read depths between high and low expression alleles in cDNA (Fig. 1, Mann-Whitney U test: W = 4537, p < 0.001), but notably, this difference in relative read depths between alleles in cDNA was not found in gDNA (Fig. 1, Mann-Whitney U test: W = 2423, p = 0.6985), excluding that biased PCR amplification was driving the observed pattern. On average 3 ± 1 alleles per individual had high expression (minimum two alleles and maximum five) and 6 ± 2 had low expression (minimum two alleles and maximum ten).

**Figure 2:**
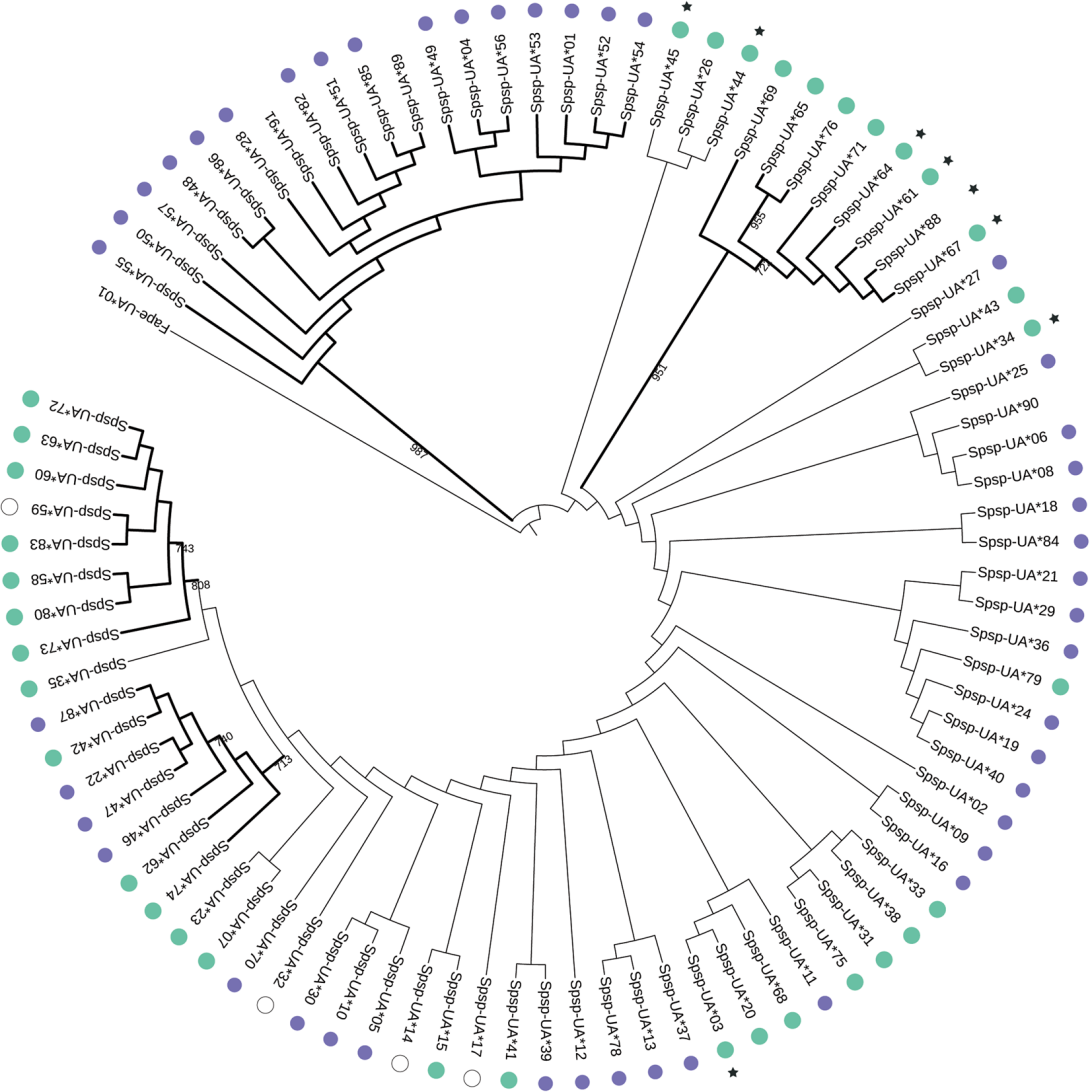
Maximum likelihood tree based on 88 MHC class I exon 3 nucleotide sequences from siskins. One MHC class I sequence (GenBank acc. nr. JN613264) from *Falco peregrinus* was used as the outgroup. The tree was constructed with PhyML software (version 3.1.2) using the K80 model with gamma distribution and 1000 bootstraps, displaying bootstraps values higher than 700. Four clusters had high bootstrap support which are indicated with bold lines. Green circles represent alleles with high expression, purple circles represent alleles with low expression, white circles represent alleles with undetermined expression and alleles without circles are not expressed. Stars (*) indicates alleles that had a 3 bp insertion. Branch length is unscaled, *i*.*e*. all branches are the same length. All non-classical alleles (N = 18) are found in one cluster with strong bootstrap support (987).

A phylogenetic reconstruction of the 88 gDNA MHC-I alleles revealed four clusters with high bootstrap support, though the majority of the clusters had low support (Fig. 2, Supplementary Fig. 5). Alleles with high or low expression were spread across the tree but two of the four highly supported clusters only contained high expression alleles. The third cluster contained both high and low expression alleles. Interestingly, the forth cluster (bootstrap support 987) contained 16 alleles with low expression and two alleles that were not expressed. These alleles fulfilled the criteria that we here used to define putatively non-classical alleles and although we cannot at this point determine if these alleles are truly non-classical by estimating their expression level on the cell surface or determine which receptors they interact with they are out of convenience hereafter called ‘non-classical alleles’. Finally, alleles that were not expressed (N = 4) were spread across the tree and alleles with a 3 bp insertion (N = 8) were found in three different clusters, suggesting that these features have rather weak phylogenetic signals.

### Variation in gene expression of classical and non-classical MHC-I alleles

**Figure 3:**
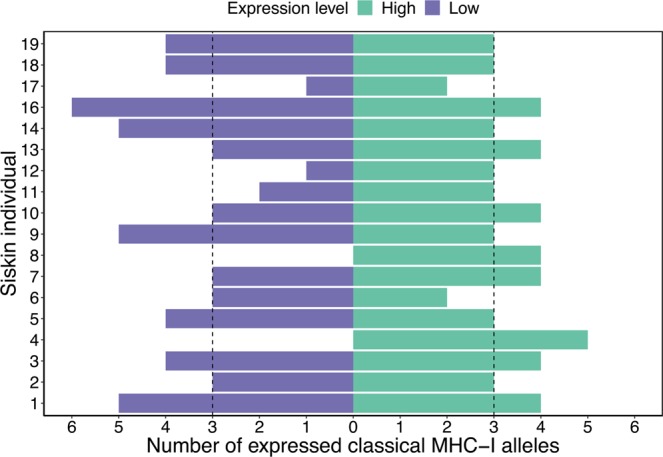
Number of expressed classical alleles in 18 siskin individuals. Green indicates alleles that are highly expressed and purple alleles with low expression, the dotted line indicates the average number of expressed alleles in each group.

All individuals had both classical and non-classical MHC-I alleles and siskins had a higher number of classical than non-classical alleles per individual (classical 8 ± 2: minimum four alleles and maximum 12; non-classical 3 ± 1: minimum two alleles and maximum six), and the majority of both types of alleles were expressed (classical 7 ± 2: minimum three alleles and maximum 10 alleles; non-classical 3 ± 1: minimum two alleles and maximum six). In gDNA, classical alleles had lower variation in relative read depth compared to non-classical alleles (Supplementary Fig. 6, Flinger-Killen: χ2 (1) = 12.959, p < 0.001), but in cDNA classical alleles had higher variation in relative read depth compared to non-classical (Supplementary Fig. 6, Flinger-Killen: χ2 (1) = 47.819, p < 0.001). The low variation in relative read depth in cDNA for non-classical alleles was expected because one of the criteria defining non-classical alleles was low expression. The large variation in relative read depth for classical alleles shows that they have either high or low expression. Sixty-four out of 68 expressed classical alleles could be sorted into high or low expression groups (35 high and 29 low). The average number of high expression classical alleles per individual was 3 ± 1 (minimum two expressed alleles and maximum five) and the average number of low expression classical alleles was similar, 3 ± 2 (minimum zero expressed alleles and maximum six) (Fig. 3).

We found 23 MHC-I alleles which were identified in more than one individual and 22 out of these 23 alleles had identical gene expression levels, *i*.*e*. high or low expression, independent of individual. However, the expression of one MHC-I allele (Spsp-UA*59) differed between individuals and a likely explanation is that this particular allele was found in two different gene copies (at two loci), one with high and one with low expression (Supplementary Table 1).

### Divergence of classical and non-classical MHC-I alleles

**Figure 4:**
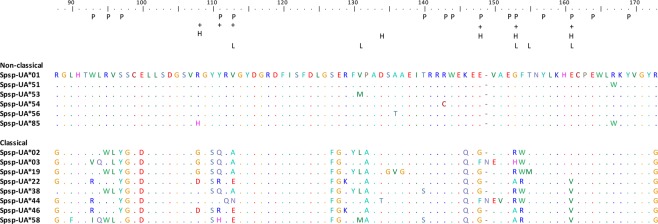
Alignment of amino acid sequences from both non-classical and classical siskin MHC-I alleles. The six non-classical alleles represent the six unique amino acids found whereas the eight classical alleles were chosen to represent the large variation among classical alleles. The amino acid positions are based on a chicken MHC-I BF2 allele and P indicates sites that belong to the peptide binding region (PBR), both are inferred from Karlsson and Westerdahl (2013). Positively selected sites from CODEML analyses using four different data sets are shown: non-classical alleles (N = 13, no positively selected sites), classical alleles (N = 54, six positively selected sites (+)), highly expressed classical alleles (N = 30, five positively selected sites (H)), low expression classical alleles (N = 22, five positively selected sites (L)).

The non-classical alleles had lower sequence divergence than the classical alleles (Table 1). The haplotype degeneracy, measured as the ratio between number of amino acid alleles and number of nucleotide alleles, was considerably lower in non-classical alleles than in classical alleles, as was the difference in P-distance (Table 1). Positively selected sites in MHC genes are frequent in classical MHC genes but rare in non-classical genes. We found six positively selected sites in the classical alleles and none in the non-classical alleles (Fig. 4), a pattern that remained the same when using equal numbers of classical and non-classical alleles (Supplementary Table 2). The rate of synonymous substitutions (dS) was a tenfold higher in classical alleles compared to non-classical alleles (0.048 in non-classical alleles and 0.126 in classical), though the rate of non-synonymous substitutions (dN) was one hundredfold higher in classical alleles (0.005 in non-classical alleles, 0.100 in classical alleles). The difference in magnitude for non-synonymous substitutions between classical and non-classical alleles is likely to be explained by different selection pressures, where classical genes are subjected to balancing selection whereas purifying selection is dominating for non-classical genes. The difference in rate of synonymous substitutions between classical and non-classical alleles could be due to hitchhiking *i.e.* the high rate of non-synonymous substitutions in classical alleles will also increase the number of synonymous substitutions. There were only minor differences in sequence divergence when comparing classical high expression and low expression alleles, whereas there was a large difference in sequence divergence when comparing classical and non-classical alleles (Table 1). These findings suggest that all putatively classical alleles, regardless of expression level, are likely to be classical. Finally, there are several unique motifs in the non-classical alleles that are not found in classical alleles (Fig. 4, for all alleles see Supplementary Fig. 3). Seven sites were conserved and unique to non-classical alleles (98 S, 113 V, 128E, 146 K, 148E, 154 F, 173 R) whereas only two sites were conserved and unique to classical alleles (146Q, 173 G).

**Table 1:** The sequence divergence of classical and non-classical MHC-I alleles in siskins was estimated using several measurements: number of nucleotide alleles (NA), number of amino acid alleles (AA), haplotype degeneracy (measured as the ratio between AA and NA), sequence divergence based on amino acid P-distance and number of positively selected sites.

	Nucleotide alleles (NA)	Amino acid alleles (AA)	Ratio AA to NA	P-distance	S.E.	Positively selected sites
Non-classical alleles	13 (18)	5	0.38	0.011	0.005	0
Classical alleles all	54 (70)	50	0.93	0.163	0.028	6
*i) Classical alleles high expression*	*30 (37)*	29	*0*.*97*	*0*.*180*	*0*.*028*	5
*ii) Classical alleles low expression*	*22 (27)*	19	*0*.*86*	*0*.*093*	*0*.*021*	5

Note that all analyses of divergence were carried out on unique nucleotide alleles that had been trimmed to have the same length, *i*.*e*. 56 or 57 amino acid long sequences (original amplicon lengths varied between 76 and 86 amino acids depending on primer combination), therefore the N-values are lower than the total number of classical and non-classical alleles identified. The number of nucleotide alleles refers to the number of unique alleles that were used in the analysis and in brackets is the total number of alleles identified when the original allele lengths were considered. The sequence divergence was also calculated separately for classical alleles with high expression and for classical alleles with low expression, excluding the two classical alleles that were not expressed and the four classical alleles with undetermined expression.

## Discussion

In common with most other passerine birds, siskins have highly duplicated MHC-I genes and in our study we found between six and 16 different MHC-I alleles per individual and the majority (95%) of these alleles were expressed. Interestingly, siskins do not have a single highly expressed MHC-I gene as has been reported and considered to be the standard among a large number of different bird species from several different bird orders (Passeriformes: the house sparrow, the tree sparrow^33^; Galliformes: the chicken^39–41^, the turkey^43^, the Japanese quail^37^; Anseriformes: the mallard^42^). On the contrary, siskins have at least three highly expressed MHC-I gene copies as indicated by up to five highly expressed MHC-I alleles. Note, that we only have looked at gene expression on the RNA level in the present study and it remains to be confirmed that this pattern also holds true for the expression of MHC molecules on the cell surface.

In the chicken, the turkey, the Japanese quail and the mallard, the dominantly expressed MHC-I gene is located next to the TAP genes^37,42–44^ and it is thought that the co-evolution between TAP and MHC-I has resulted in a single highly expressed MHC-I gene copy^51^. On the contrary, in mammals TAP is not located near the MHC-I genes and in mammals there is no evidence for a dominantly expressed MHC-I gene copy^52^. There is however evidence that the three classical genes (HLA -A, -B, -C) are expressed to different degrees, HLA-C has lower expression than HLA -A and -B^53^. To date we do not know the location of TAP in passerines but there is evidence from the zebra finch genome that the MHC-I genes and TAP genes are unlikely to be located next to each other^49,50^. Hence, one possible explanation for the higher number of highly transcribed MHC-I gene copies in siskins could be that the connection between MHC-I and TAP has been lost.

Most passerine birds have high MHC-I diversity, measured as the number of different MHC-I alleles per individual ^*e.g.*^^8,11,47,48^. Several different mechanisms can explain how the large number of MHC-I gene copies are generated, *i*.*e*. whereby one MHC-I gene copy eventually results in a large number of different MHC-I gene copies. One option could be that transposable elements trigger the initiation of gene duplication where after ectopic recombination during the meiosis furthers the duplication events on one of the haplotypes^54^. Gene copies can then recombine further and the number of gene copies per haplotype increases, see evolution of gene families ^*e.g.*^^1,2^. This process is likely to result in gene copy number variation between individuals and also between haplotypes within individuals. An interesting finding in chickens, that potentially could explain some parts of the observed high MHC diversity in passerines, is that the number of microchromosomes can vary between individuals^55,56^. Trisomy of microchromosomes, carrying three chromosomes, does not infer strong negative effects in chickens and since the MHC region is found on a microchromosome (chromosome 16) trisomy can increase the MHC diversity within an individual in chickens. MHC-I gene copy number variation between haplotypes, trisomy and degree of homozygosity can explain odd numbers of alleles in gDNA within individuals. In our siskin data even and odd numbers of MHC-I alleles per individual were equally common.

The majority of the MHC-I alleles that are characterized in passerines to date are in open reading frame which indicates that they are likely to be expressed ^*e.g.*^^8,11,47,48^. The selection for maintaining an open reading frame is lost in genes that are not expressed and such genes become pseudogenized^57^. We therefore envision that a large proportion of the number of MHC-I gene copies that is found in passerines is actively used and that there is an on-going selection for maintaining a high number of MHC-I gene copies per individual. Though, it should be mentioned that most studies on passerines have only DNA sequenced exon 3, *i*.*e*. 300 bp that encode the most variable part of the MHC-I gene. It is likely that a subset of these ‘exon 3 alleles’ would have been identified as pseudogenes if the full genes had been DNA sequenced. Interestingly, the four studies that have examined expression of MHC-I genes in passerine birds to date have found that a considerable proportion of the MHC-I alleles are expressed (transcribed)^33,58,59^.

In a previous study Drews *et al*.^33^ reported expression patterns of MHC-I alleles in three species of sparrows; house sparrow, tree sparrow and Spanish sparrow (*Passer hispaniolensis*), as well as the relative MHC-I expression in two of these species (house sparrow and tree sparrow). The putatively classical MHC-I alleles in sparrows were expressed to both high and low degrees, in line with our findings in the siskins^33^. However, siskins have a larger number of classical MHC-I alleles that are highly expressed than sparrows (siskin: 5, house sparrow: 2, tree sparrow: 2). Interestingly, this difference in the number of highly expressed classical MHC-I alleles cannot be explained by the number of classical MHC-I gene copies in the genome (gDNA alleles), since siskins and tree sparrows have similar numbers of classical gDNA alleles (siskin: 8, tree sparrow: 10). However, siskins express a larger proportion of their classical MHC-I alleles than sparrows (siskin: 97%, average 7 alleles expressed; house sparrow: 61%, average 3 alleles expressed; Spanish sparrow: 45%, average 3 alleles expressed; tree sparrow: 41%, average 4 alleles expressed). The number of expressed classical MHC-I alleles vary between individuals, in both siskins and sparrows, and an odd number of highly expressed alleles are as common as an even number. At first thought an odd number of highly expressed alleles is counter intuitive given that we expect each gene copy to have either high or low expression. Moreover, since most MHC-I genes are expected to be heterozygote, given their high MHC polymorphism, we expect even numbers of highly expressed alleles. Gene copy number variation between haplotypes within individuals, varying levels of homozygosity and the putative possibility of trisomy are mechanisms that can explain odd numbers of highly expressed genes.

Why then, do siskins have a larger number of highly expressed classical MHC-I gene copies than sparrows? One explanation could be that sparrows only express half the number of MHC-I alleles compared with siskins, hence they have fewer gene copies that can be highly expressed. Also, the sample size of siskins is three times larger than for the sparrow species combined and there are hence more opportunities to detect variance between individuals in gene expression in siskins. It is worth mentioning that two out of 18 siskins only had two highly expressed MHC-I alleles, which could indicate the existence of a single highly expressed MHC-I gene copy. Moreover, homozygosity and heterozygosity may vary between siskins and sparrows and if sparrows are more homozygous then two highly expressed MHC-I alleles actually can mean two highly expressed MHC-I gene copies. Finally, sparrows and siskins can be subject to different selective regimes from pathogens, selecting for different optimal levels of MHC-I diversity.

Classical and non-classical MHC-I genes have been reported in a wide range of different vertebrates, and it seems likely that both kinds of genes occur frequently ^*e.g.*^^31,60–62^. However, there are many different types of non-classical genes, for example the non-classical MHC-I genes (HLA -E, -F, -G) in humans have high sequences similarities with classical genes (HLA -A, -B, -C), although the encoded MHC molecules have rather different immune functions^4,22^. Other non-classical human MHC genes, such as the MIC -A and -B genes, have evolved more diverged functions and they no longer interact with T-cells^4^. The MIC genes have considerably lower sequence similarities with the HLA -A, -B, -C genes than the HLA- E, -F, -G genes do^63^. In chicken, alleles from a single MHC-Y gene have considerably higher sequence similarity (93% similarity between the alleles within this non-classical gene) than with alleles from the MHC-B genes (73% similarity between classical and non-classical alleles)^20^. The MHC-Y alleles are expressed on the cell surface but changes in the amino acids in the peptide binding cleft indicates that they bind to a different type of antigens compared to their classical counterparts^20^. The putatively non-classical genes in siskins were inferred based on the fact that we found one group of low expressed alleles that formed a highly supported cluster in the phylogenetic tree containing all identified siskin MHC-I alleles. These putatively non-classical alleles had much lower sequences divergence compared to the alleles in the rest of the tree. Putatively non-classical MHC-I alleles in sparrows show identical patterns^33^. However, with the data available from siskins and sparrows so far it cannot be ruled out that the non-classical alleles are low expressed pseudogenes or that the high similarity is because all these alleles stem from a recent duplication event, hence more studies are still needed in order to more fully confirm that the putatively non-classical alleles in these passerine species indeed are non-classical. It is noteworthy that although, both siskins and sparrows have putatively non-classical genes these genes seem to have different evolutionary origins. The most recent common ancestor of the house sparrow, Spanish sparrow and tree sparrow was seven million years ago and the non-classical genes in these sparrows all stem from a shared common ancestor^33^. Similarly, among the galliforms, chicken and turkey had a common ancestor 28–40 million years ago, and their non-classical MHC genes (MHC-Y) also have a shared common ancestor^38,64,65^. Siskins and sparrows have a common ancestor 29 million years ago^66,67^, but there is no evidence that their non-classical MHC-I alleles have a common ancestry (Supplementary Fig. 7).

## Conclusion

In contrast to previous findings of a single dominantly expressed classical MHC-I gene in birds of the orders Galliformes and Anseriformes, and also in sparrows of the order Passeriformes, siskins have as many as five highly expressed classical MHC-I alleles. High expression in combination with signs of positive selection strongly suggests that these putatively classical MHC-I genes play a key role in the adaptive immunity. Moreover, we identified putatively non-classical MHC-I alleles in one additional passerine species, the siskin, suggesting that non-classical genes are likely to be a common feature also among birds of the order Passeriformes. The occurrence of classical and non-classical genes partly explains the high MHC-I diversity in passerines, though what matters for antigen presentation is really the expressed MHC-I divergence and this area is to a large degree still unknown in passerines.

## Methods

### Sample collection

Wild juvenile siskins were caught with mist nets in June 2013 on the Curonian Spit in the Baltic Sea (Kaliningrad region, Russia) and were then housed in aviaries at the Biological Station Rybachy of the Zoological Institute of the Russian Academy of Sciences. Blood samples (10 μl) were taken from the brachial vein. For DNA samples heparinized microcapillaries was used and the blood was stored in SET-buffer and stored at −40 °C until extraction. For the RNA samples, the blood was immediately frozen in liquid nitrogen and stored at −80 °C until extraction.

### DNA and RNA extraction

DNA was extracted using a standard ammonium-acetate protocol^68^. RNA was extracted using a combination of the TRIzol LS manufacturer protocol (Life Technologies) and the RNeasy Mini kit (QIAGEN), see Drews *et al*.^33^ for details, including a DNase treatment in order to remove any gDNA contamination. In order to confirm that the DNase treatment was successful the RNA was used as a template in a standard PCR reaction which resulted in no amplification. The RNA was reverse transcribed to complementary DNA (cDNA) using the RETROscript kit (Life Technologies) according to the manufacturer’s protocol.

### Sequencing of exon 2-4 and primer design

Since no MHC-I sequences from siskin have previously been published five different primer combinations from other songbird species were initially used to amplify MHC-I exon 2-4 in one siskin individual (primer location can be found in Supplementary Fig. 8, annealing temperatures and primer sequences in Supplementary Table 3, details on methods in Supplementary methods). In total four verified alleles were obtained and these were aligned with MHC alleles from related species (Atlantic canary (XM_018925088.1), the house finch (KC585535.1), the common rosefinch (JN713104), the collared flycatcher (XM_016305376.1) and the lark sparrow (KF803770.1)). Based on this alignment four new primers that amplified exon 3 in siskins were designed.

### Illumina MiSeq sequencing of exon 3

In order to determine the number of MHC-I exon 3 alleles, as well as the number of MHC-I alleles that were expressed we sequenced gDNA and cDNA samples from 19 siskins with Illumina amplicon sequencing. After initial testing, three primer combinations were chosen and used for the Illumina amplicon sequencing (primer locations can be found in Supplementary Fig. 8, annealing temperatures and primer sequences in Supplementary Table 3). The final library was sent for 300 bp paired-end Illumina MiSeq sequencing at the DNA sequencing facility, Department of Biology, Faculty of Science, Lund University. In total 90 samples were sequenced (19 gDNA + 4 duplicates for primer combination 1, 19 gDNA samples + 4 duplicates + 19 cDNA samples + 3 duplicates for primer combination 2 and 19 cDNA samples and 3 duplicates for primer combination 3). For details on library preparation see Supplementary methods.

### Filtering of Illumina MiSeq data

After the Illumina sequencing the reads were processed separately for each primer combination and DNA type (*i*.*e*. gDNA and cDNA). The sequencing failed for two samples (the cDNA samples from individual 15 with both primer combination 2 and 3) and hence this individual was removed from any further analysis. After initial processing where the primer sequences were removed the raw reads where further processed using the DADA2 program^69^. During this process, low quality reads were removed, errors were re-assign to the parental sequence from which they arose (*i*.*e*. read clustering) and finally the forward and reverse reads were merged^69^. Reads that deviated from the expected length, except for those which occurred in multiples of three bases and thus did not alter the reading frame, were removed. For the gDNA samples a minimum per amplicon frequency threshold, based on the repeatability of the duplicated samples, was set for each primer combination and all sequences remaining in the data set after this point was considered true alleles. For the cDNA samples, the data was compared to the gDNA data and any allele that was found in both sets were considered to be expressed. In the case when cDNA sequences were not found in the gDNA data, a minimum per amplicon frequency threshold was set based on the average read depth for each primer combination and requiring a minimum of 100 reads per allele. All sequences remaining in the cDNA data after these filtering steps were considered true expressed alleles. For details on filtering see Supplementary methods.

### Determining expression

The expressed alleles (N = 84) were divided into two groups depending on whether they had high or low expression. The threshold for determining if an allele was highly expressed was set specifically for each individual and primer combination (primer combination 2: that amplify within exon 3 and primer combination 3: that amplify from exon 2 to exon 3). An allele was considered to be highly expressed if it had a higher relative read depth than expected given that all alleles were expressed equally. 59 out of 84 alleles were amplified with both primer combinations and here the expression levels were compared between the primer combinations within individual, (Supplementary Table 1). The two primer combinations agreed to 92% (N = 119 comparison: 45 high expression, 74 low expression) but in 11 comparisons they did not. These discrepancies could be caused by primer amplification preferences, poor amplification or allelic dropout. In order to try to solve this discrepancy addition rules were applied. First, we took advantage of the fact that primer combination 2 had been used on both gDNA and cDNA samples and compared the relative read depth (Supplementary Fig. 4). An allele was determined as highly expressed if the relative read depth was clearly higher in the cDNA sample compared to the gDNA sample, the relative read depth in the cDNA sample needed to be one forth higher than the relative read depth in the gDNA sample (Supplementary Fig. 9). To further verify that this was a good measurement the relative read depth in gDNA and cDNA was compared for all alleles that had been identified as highly expressed with primer combination 2 (N = 49 comparisons) and the two measurements agreed to 100%. The rule that the relative read depth in the cDNA sample needed to be one forth higher than the relative read depth in the gDNA sample identified that four out of the 11 comparison showed high expression. One more rule was applied in order to determine the remaining discrepancies, and this rule was more of a cleaning step where any allele that had just barely been amplified in the cDNA sample and also not been amplified satisfactory in the gDNA sample was considered an amplification error and removed. This resolved the expression level for four more comparisons. This left three out of 11 comparisons (three different alleles) where the expression level could not be determined. Finally, the expression level was compared across individuals and the majority of alleles that had been amplified in more than one individual had identical gene expression levels (N = 22 out 24 alleles). One of the two alleles that indicated different expression levels was determined as highly expressed since it clearly had high expression in one individual and in the other individual the expression level was just barley under the limit of being classified as highly expressed. For the other allele the expression varied to much (indicated as high in six individuals and as low in 6 individuals) hence the expression for this allele could not be determined. In total the degree of expression could not be determined for 4 alleles.

### Data analysis

A maximum likelihood tree based on all verified siskin MHC-I exon 3 alleles was constructed using the PhyML software v 3.1.2. The K80 model with gamma distribution was specified as recommended by jModelTest v 2.1.7 with 1000 bootstraps and illustrated with iTOL v 3.4.3 using an MHC-I exon 3 allele from peregrine falcons as the outgroup (GenBank acc. nr. JN613264.1). All statistical analyses were performed in R v 2.15.3^70^. Mann-Whitney U tests were used to compare the amplification of high and low expressed alleles in gDNA and cDNA, respectively. Flinger-Killen test of homogeneity of variances was used to determine the difference in expression between non-classical and classical alleles. Plots were produced using ggplot2 in R^71^. To test for recombination the GARD method^72^ was used trough the web application datamonkey^73–75^ (http://classic.datamonkey.org), and no evidence of recombination was found. Positively selected sited were determined with the CODEML program in the PAML package v 4.8^76,77^. The rate of non-synonymous sites (dN) and synonymous sites (dS) as well as P-distances were calculated in MEGA 7^78^.

## Supplementary information

Supplementary information
