# Supplementary material for: Not all birds have a single dominantly expressed MHC-I gene: Transcription suggests that siskins have many highly expressed MHC-I genes
Source: Sci Rep. 2019 Dec 20;9:19506. doi: 10.1038/s41598-019-55800-9 (PMC6925233; doi:10.1038/s41598-019-55800-9)
Supplement: Supplementary file 1 — Supplementary information [file 41598_2019_55800_MOESM1_ESM.pdf]

Not all birds have a single dominantly expressed MHC-I gene: Transcription suggests that siskins have many highly expressed MHC-I genes

Anna Drews[1,*] and Helena Westerdahl[1]

[1] Department of Biology, Lund University, Lund, Sweden.

[*] Corresponding author: anna.drews@biol.lu.se

Supplementary Table 1: Comparison of the three primer combinations used to amplify MHC-I in siskins. X indicates an allele that have been amplified in gDNA. The expression has been determined based on the relative read depth of each allele within individual and H indicates an allele that have been identified as highly expressed, L alleles that have been identified as having low expression and H* indicates allele that have been determine to have high expression after corrections.

Allele	Individual 1				Individual 2				Individual 3			
	Primer combination 1 (gDNA)	Primer combination 2 (gDNA)	Primer combination 2 (cDNA)	Primer combination 3 (cDNA)	Primer combination 1 (gDNA)	Primer combination 2 (gDNA)	Primer combination 2 (cDNA)	Primer combination 3 (cDNA)	Primer combination 1 (gDNA)	Primer combination 2 (gDNA)	Primer combination 2 (cDNA)	Primer combination 3 (cDNA)
Spsp-UA*01	X	X	L	L	X	X	L	L	X	X	L	L
Spsp-UA*02	X	X	L	L	0	0	0	0	0	0	0	0
Spsp-UA*03	0	0	0	0	X	X	H	H	0	0	0	0
Spsp-UA*04	X	0	0	L	0	0	0	0	X	0	0	L
Spsp-UA*05	0	0	0	0	0	0	0	0	0	0	0	0
Spsp-UA*06	X	0	0	L	0	X	L	0	X	0	0	L
Spsp-UA*07	0	0	0	0	0	0	0	0	0	0	0	0
Spsp-UA*08	0	0	0	0	0	0	0	0	0	0	0	0
Spsp-UA*09	0	0	0	0	0	0	0	0	0	0	0	0
Spsp-UA*10	0	0	0	0	0	0	0	0	0	0	0	0
Spsp-UA*11	0	0	0	0	0	0	0	0	X	X	L	L
Spsp-UA*12	0	0	0	0	0	0	0	0	0	0	0	0
Spsp-UA*13	0	0	0	0	0	0	0	0	0	0	0	0
Spsp-UA*14	0	0	0	0	0	0	0	0	0	0	0	0
Spsp-UA*15	0	0	0	0	0	0	0	0	0	0	0	0
Spsp-UA*16	0	0	0	0	0	0	0	0	0	0	0	0
Spsp-UA*17	0	0	0	0	0	0	0	0	0	0	0	0
Spsp-UA*18	0	0	0	0	0	0	0	0	0	0	0	0
Spsp-UA*19	0	0	0	0	0	0	0	0	0	0	0	0
Spsp-UA*20	0	0	0	0	0	0	0	0	0	0	0	0
Spsp-UA*21	0	0	0	0	0	0	0	0	0	0	0	0
Spsp-UA*22	X	X	L	L	0	0	0	0	0	0	0	0
Spsp-UA*23	0	0	0	0	0	0	0	0	0	0	0	0
Spsp-UA*24	0	0	0	0	0	0	0	0	0	0	0	0
Spsp-UA*25	0	0	0	0	X	X	L	L	0	0	0	0
Spsp-UA*26	0	0	0	0	0	0	0	0	0	0	0	0
Spsp-UA*27	0	0	0	0	0	0	0	0	0	0	0	0
Spsp-UA*28	0	0	0	0	X	X	L	L	X	X	L	L
Spsp-UA*29	0	0	0	0	0	0	0	0	0	0	0	0
Spsp-UA*30	0	0	0	0	0	0	0	0	0	0	0	0
Spsp-UA*31	0	0	0	0	0	0	0	0	0	0	0	0
Spsp-UA*32	0	0	0	0	0	0	0	0	0	0	0	0
Spsp-UA*33	0	0	0	0	0	0	0	0	X	X	H	H
Spsp-UA*34	0	0	0	0	0	0	0	0	0	0	0	0
Spsp-UA*35	0	0	0	0	0	0	0	0	0	0	0	0
Spsp-UA*36	0	0	0	0	0	0	0	0	X	X	L	L
Spsp-UA*37	X	X	L	L	0	0	0	0	0	0	0	0
Spsp-UA*38	0	0	0	0	0	0	0	0	0	0	0	0
Spsp-UA*39	X	X	L	L	0	0	0	0	0	X	L	0
Spsp-UA*40	0	0	0	0	0	0	0	0	0	0	0	0
Spsp-UA*41	X	X	H	H	0	0	0	0	0	0	0	0
Spsp-UA*42	0	0	0	0	0	0	0	0	0	0	0	0
Spsp-UA*43	X	0	0	H	0	0	0	0	0	0	0	0
Spsp-UA*44	0	0	0	0	0	0	0	0	0	0	0	0
Spsp-UA*45	0	0	0	0	0	0	0	0	0	0	0	0
Spsp-UA*46	0	0	0	0	0	0	0	0	0	0	0	0
Spsp-UA*47	0	0	0	0	0	0	0	0	0	0	0	0
Spsp-UA*48	0	0	0	0	0	0	0	0	0	0	0	0
Spsp-UA*49	0	0	0	0	0	0	0	0	0	0	0	0
Spsp-UA*50	0	0	0	0	X	X	L	L	0	0	0	0
Spsp-UA*51	0	0	0	0	0	0	0	0	0	0	0	0
Spsp-UA*52	0	0	0	0	0	0	0	0	0	0	0	0
Spsp-UA*53	0	0	0	0	0	0	0	0	0	0	0	0
Spsp-UA*54	0	0	0	0	X	X	L	L	0	0	0	0
Spsp-UA*55	0	0	0	0	0	0	0	0	0	0	0	0
Spsp-UA*56	0	0	0	0	0	0	0	0	0	0	0	0
Spsp-UA*57	0	0	0	0	0	0	0	0	X	X	L	L
Spsp-UA*58	0	X	H	H	0	X	H	H	0	X	H	H
Spsp-UA*59	0	0	0	0	0	X	H	H	0	0	L	0
Spsp-UA*60	0	0	0	0	0	0	0	0	0	0	0	0
Spsp-UA*61	0	0	0	0	0	0	0	0	0	X	H	H
Spsp-UA*62	0	0	0	0	X	X	H*	H	0	0	0	0
Spsp-UA*63	0	0	0	0	0	0	0	0	0	0	0	0
Spsp-UA*64	0	0	0	0	0	0	0	0	0	0	0	0
Spsp-UA*65	0	0	0	0	0	0	0	0	0	0	0	0
Spsp-UA*67	0	0	0	0	0	0	0	0	0	0	0	0
Spsp-UA*68	0	X	H	0	0	0	0	0	0	0	0	0
Spsp-UA*69	0	0	0	0	0	0	0	0	0	0	0	0
Spsp-UA*70	0	0	0	0	0	0	0	0	0	0	0	0
Spsp-UA*71	0	0	0	0	0	0	0	0	0	0	0	0
Spsp-UA*72	0	0	0	0	0	0	0	0	0	X	H	H
Spsp-UA*73	0	0	0	0	0	0	0	0	0	0	0	0
Spsp-UA*74	0	0	0	0	0	0	0	0	0	0	0	0
Spsp-UA*75	0	0	0	0	0	0	0	0	0	0	0	0
Spsp-UA*76	0	0	0	0	0	0	0	0	0	0	0	0
Spsp-UA*78	0	0	0	0	0	0	0	0	0	0	0	0
Spsp-UA*79	0	0	0	0	0	0	0	0	0	0	0	0
Spsp-UA*80	0	0	0	0	0	0	0	0	0	0	0	0
Spsp-UA*82	0	0	0	0	0	0	0	0	0	0	0	0
Spsp-UA*83	0	0	0	0	0	0	0	0	0	0	0	0
Spsp-UA*84	0	0	0	0	0	0	0	0	0	0	0	0
Spsp-UA*85	0	0	0	0	0	0	0	0	0	0	0	0
Spsp-UA*86	0	0	0	0	0	0	0	0	0	0	0	0
Spsp-UA*87	0	0	0	0	0	X	L	0	0	0	0	0
Spsp-UA*88	0	0	0	0	0	0	0	0	0	0	0	0
Spsp-UA*89	0	0	0	0	0	0	0	0	0	0	0	0
Spsp-UA*90	0	0	0	0	0	0	0	0	0	0	0	0
Spsp-UA*91	0	0	0	0	0	0	0	0	0	0	0	0

Allele	Individual 4				Individual 5				Individual 6			
	Primer combination 1 (gDNA)	Primer combination 2 (gDNA)	Primer combination 2 (cDNA)	Primer combination 3 (cDNA)	Primer combination 1 (gDNA)	Primer combination 2 (gDNA)	Primer combination 2 (cDNA)	Primer combination 3 (cDNA)	Primer combination 1 (gDNA)	Primer combination 2 (gDNA)	Primer combination 2 (cDNA)	Primer combination 3 (cDNA)
Spsp-UA*01	X	X	L	L	X	X	L	L	X	X	L	L
Spsp-UA*02	0	0	0	0	0	0	0	0	X	X	L	L
Spsp-UA*03	0	0	0	0	0	0	0	0	0	0	0	0
Spsp-UA*04	X	0	0	L	X	0	0	L	X	0	0	L
Spsp-UA*05	0	0	0	0	0	0	0	0	0	0	0	0
Spsp-UA*06	0	0	0	0	0	0	0	0	0	0	0	0
Spsp-UA*07	0	0	0	0	0	0	0	0	0	0	0	0
Spsp-UA*08	0	0	0	0	0	0	0	0	0	0	0	0
Spsp-UA*09	0	0	0	0	0	0	0	0	0	0	0	0
Spsp-UA*10	0	0	0	0	0	0	0	0	0	0	0	0
Spsp-UA*11	0	0	0	0	0	0	0	0	0	0	0	0
Spsp-UA*12	0	0	0	0	0	0	0	0	X	X	L	L
Spsp-UA*13	0	0	0	0	X	X	L	0	0	0	0	0
Spsp-UA*14	0	0	0	0	0	0	0	0	X	X	L	H
Spsp-UA*15	0	0	0	0	0	0	0	0	0	0	0	0
Spsp-UA*16	0	0	0	0	X	X	0	0	0	0	0	0
Spsp-UA*17	0	0	0	0	0	0	0	0	0	0	0	0
Spsp-UA*18	0	0	0	0	X	X	L	L	0	0	0	0
Spsp-UA*19	0	0	0	0	0	0	0	0	0	0	0	0
Spsp-UA*20	0	0	0	0	0	0	0	0	0	0	0	0
Spsp-UA*21	0	0	0	0	0	0	0	0	0	0	0	0
Spsp-UA*22	0	0	0	0	X	X	L	L	0	0	0	0
Spsp-UA*23	0	0	0	0	0	0	0	0	0	0	0	0
Spsp-UA*24	0	0	0	0	0	0	0	0	0	0	0	0
Spsp-UA*25	0	0	0	0	0	0	0	0	0	0	0	0
Spsp-UA*26	0	0	0	0	0	0	0	0	0	0	0	0
Spsp-UA*27	0	0	0	0	0	0	0	0	0	0	0	0
Spsp-UA*28	0	X	L	L	X	X	L	L	0	0	0	0
Spsp-UA*29	0	0	0	0	0	0	0	0	0	0	0	0
Spsp-UA*30	0	0	0	0	0	0	0	0	0	0	0	0
Spsp-UA*31	0	0	0	0	X	X	H	H	0	0	0	0
Spsp-UA*32	0	0	0	0	0	0	0	0	0	0	0	0
Spsp-UA*33	0	0	0	0	0	0	0	0	0	0	0	0
Spsp-UA*34	0	0	0	0	0	0	0	0	0	0	0	0
Spsp-UA*35	0	0	0	0	0	0	0	0	0	0	0	0
Spsp-UA*36	0	0	0	0	0	0	0	0	0	0	0	0
Spsp-UA*37	0	0	0	0	0	0	0	0	0	0	0	0
Spsp-UA*38	0	0	0	0	0	0	0	0	0	0	0	0
Spsp-UA*39	0	0	0	0	0	0	0	0	0	0	0	0
Spsp-UA*40	0	0	0	0	0	0	0	0	0	0	0	0
Spsp-UA*41	0	0	0	0	0	0	0	0	0	0	0	0
Spsp-UA*42	0	0	0	0	0	0	0	0	0	0	0	0
Spsp-UA*43	0	0	0	0	0	0	0	0	0	0	0	0
Spsp-UA*44	0	0	0	0	0	0	0	0	0	0	0	0
Spsp-UA*45	0	0	0	0	X	X	H	H	0	0	0	0
Spsp-UA*46	0	0	0	0	0	0	0	0	X	X	L	L
Spsp-UA*47	0	0	0	0	0	0	0	0	0	0	0	0
Spsp-UA*48	0	0	0	0	0	0	0	0	0	0	0	0
Spsp-UA*49	0	0	0	0	0	0	0	0	0	0	0	0
Spsp-UA*50	0	0	0	0	0	0	0	0	0	0	0	0
Spsp-UA*51	0	X	L	L	0	0	0	0	0	0	0	0
Spsp-UA*52	0	0	0	0	0	0	0	0	0	0	0	0
Spsp-UA*53	0	0	0	0	X	X	L	L	0	0	0	0
Spsp-UA*54	0	0	0	0	0	0	0	0	0	0	0	0
Spsp-UA*55	0	0	0	0	0	0	0	0	0	0	0	0
Spsp-UA*56	0	0	0	0	0	0	0	0	0	0	0	0
Spsp-UA*57	0	0	0	0	0	0	0	0	0	0	0	0
Spsp-UA*58	0	X	H	H	0	0	0	0	0	X	H	H
Spsp-UA*59	0	0	L	0	0	X	H	H	0	X	H	H
Spsp-UA*60	0	X	H	H	0	0	0	0	0	0	0	0
Spsp-UA*61	0	X	H	0	0	0	0	0	0	0	0	0
Spsp-UA*62	0	0	0	0	0	0	0	0	0	0	0	0
Spsp-UA*63	0	0	0	0	0	X	H	H	0	0	0	0
Spsp-UA*64	0	0	0	H	0	0	0	0	0	0	0	0
Spsp-UA*65	0	0	0	0	0	0	0	0	0	0	0	0
Spsp-UA*67	0	0	0	0	0	0	0	0	0	0	0	0
Spsp-UA*68	X	X	H	H	0	0	0	0	0	0	0	0
Spsp-UA*69	0	0	0	0	0	0	0	0	0	0	0	H
Spsp-UA*70	0	0	0	0	0	0	0	0	0	0	0	0
Spsp-UA*71	0	0	0	0	0	0	0	0	0	0	0	0
Spsp-UA*72	0	0	0	0	0	0	0	0	0	0	0	0
Spsp-UA*73	0	0	0	0	0	0	0	0	0	0	0	0
Spsp-UA*74	0	0	0	0	0	0	0	0	0	0	0	0
Spsp-UA*75	0	0	0	0	0	0	0	0	0	0	0	0
Spsp-UA*76	0	0	0	0	0	0	0	0	0	0	0	0
Spsp-UA*78	0	0	0	0	0	0	0	L	0	0	0	0
Spsp-UA*79	0	0	0	0	0	0	0	0	0	0	0	0
Spsp-UA*80	0	0	0	0	0	0	0	0	0	0	0	0
Spsp-UA*82	0	0	0	0	0	0	0	0	0	0	0	0
Spsp-UA*83	0	0	0	0	0	0	0	0	0	0	0	0
Spsp-UA*84	0	0	0	0	0	0	0	0	0	0	0	0
Spsp-UA*85	0	0	0	0	0	0	0	0	0	0	0	0
Spsp-UA*86	0	0	0	0	0	0	0	0	0	0	0	0
Spsp-UA*87	0	0	0	0	0	0	0	0	0	0	0	0
Spsp-UA*88	X	0	0	0	0	0	0	0	0	0	0	0
Spsp-UA*89	X	0	0	0	0	0	0	0	0	0	0	0
Spsp-UA*90	0	0	0	0	0	0	0	0	0	0	0	0
Spsp-UA*91	X	0	0	0	0	0	0	0	0	0	0	0

Allele	Individual 7				Individual 8				Individual 9			
	Primer combination 1 (gDNA)	Primer combination 2 (gDNA)	Primer combination 2 (cDNA)	Primer combination 3 (cDNA)	Primer combination 1 (gDNA)	Primer combination 2 (gDNA)	Primer combination 2 (cDNA)	Primer combination 3 (cDNA)	Primer combination 1 (gDNA)	Primer combination 2 (gDNA)	Primer combination 2 (cDNA)	Primer combination 3 (cDNA)
Spsp-UA*01	X	X	L	L	X	X	L	L	X	X	L	L
Spsp-UA*02	X	X	L	L	0	0	0	0	X	X	L	0
Spsp-UA*03	X	X	H	H	X	X	H	H	0	0	0	0
Spsp-UA*04	X	0	0	L	X	0	0	L	X	0	0	L
Spsp-UA*05	0	0	0	0	0	0	0	0	0	0	0	0
Spsp-UA*06	0	0	0	0	0	0	0	0	0	0	0	0
Spsp-UA*07	0	0	0	0	0	0	0	0	0	0	0	0
Spsp-UA*08	0	0	0	0	0	0	0	0	0	0	0	0
Spsp-UA*09	0	0	0	0	0	0	0	0	0	0	0	0
Spsp-UA*10	0	0	0	0	0	0	0	0	X	X	L	L
Spsp-UA*11	X	X	L	L	0	0	0	0	0	0	0	0
Spsp-UA*12	0	0	0	0	0	0	0	0	0	0	0	0
Spsp-UA*13	0	0	0	0	0	0	0	0	0	0	0	0
Spsp-UA*14	0	0	0	0	0	0	0	0	0	0	0	0
Spsp-UA*15	0	0	0	0	X	0	0	H	0	0	0	0
Spsp-UA*16	0	0	0	0	0	0	0	0	0	0	0	0
Spsp-UA*17	0	0	0	0	0	0	0	0	0	0	0	0
Spsp-UA*18	0	0	0	0	0	0	0	0	0	0	0	0
Spsp-UA*19	0	0	0	0	0	0	0	0	X	X	L	L
Spsp-UA*20	0	0	0	0	0	0	0	0	0	0	0	0
Spsp-UA*21	0	0	0	0	0	0	0	0	0	0	0	0
Spsp-UA*22	0	0	0	0	0	0	0	0	0	0	0	0
Spsp-UA*23	0	0	0	0	0	0	0	0	0	0	0	0
Spsp-UA*24	0	0	0	0	0	0	0	0	0	0	0	0
Spsp-UA*25	0	0	0	0	0	0	0	0	0	0	0	0
Spsp-UA*26	0	0	0	0	0	0	0	0	0	0	0	0
Spsp-UA*27	X	X	L	L	0	0	0	0	0	0	0	0
Spsp-UA*28	X	X	L	L	0	0	0	0	0	0	0	0
Spsp-UA*29	0	0	0	0	0	0	0	0	X	X	L	L
Spsp-UA*30	0	0	0	0	0	0	0	0	0	0	0	0
Spsp-UA*31	0	0	0	0	0	0	0	0	0	0	0	0
Spsp-UA*32	0	0	0	0	0	0	0	0	0	0	0	0
Spsp-UA*33	0	0	0	0	0	X	H*	0	0	0	0	0
Spsp-UA*34	0	0	0	0	0	0	0	0	0	0	0	0
Spsp-UA*35	0	0	0	0	0	0	0	0	0	0	0	0
Spsp-UA*36	0	0	0	0	0	0	0	0	0	0	0	0
Spsp-UA*37	0	0	0	0	0	0	0	0	0	0	0	0
Spsp-UA*38	0	0	0	0	0	0	0	0	0	0	0	0
Spsp-UA*39	0	0	0	0	0	0	0	0	0	0	0	0
Spsp-UA*40	0	0	0	0	0	0	0	0	0	0	0	0
Spsp-UA*41	0	0	0	0	0	0	0	0	0	0	0	0
Spsp-UA*42	0	0	0	0	0	0	0	0	0	0	0	0
Spsp-UA*43	0	0	0	0	0	0	0	0	0	0	0	0
Spsp-UA*44	X	X	H	H	0	0	0	0	0	0	0	0
Spsp-UA*45	0	0	0	0	0	0	0	0	0	0	0	0
Spsp-UA*46	0	0	0	0	0	0	0	0	0	0	0	0
Spsp-UA*47	0	0	0	0	0	0	0	0	0	0	0	0
Spsp-UA*48	0	0	0	0	0	0	0	0	0	0	0	0
Spsp-UA*49	0	0	0	0	0	0	0	0	0	0	0	0
Spsp-UA*50	0	0	0	0	0	0	0	0	0	0	0	0
Spsp-UA*51	0	0	0	0	0	0	0	0	0	0	0	0
Spsp-UA*52	0	0	0	0	0	0	0	0	0	0	0	0
Spsp-UA*53	0	0	0	0	0	0	0	0	0	0	0	0
Spsp-UA*54	0	0	0	0	0	0	0	0	0	0	0	0
Spsp-UA*55	0	0	0	0	0	0	0	0	0	0	0	0
Spsp-UA*56	X	X	L	L	0	0	0	0	0	0	0	0
Spsp-UA*57	0	0	0	0	0	0	0	0	0	0	0	0
Spsp-UA*58	0	X	H	H	0	X	H	H	0	X	H	H
Spsp-UA*59	0	0	L	L	0	0	0	0	0	0	0	0
Spsp-UA*60	0	0	0	0	0	0	0	0	0	0	0	0
Spsp-UA*61	0	0	0	0	0	0	0	0	0	0	0	0
Spsp-UA*62	0	0	0	0	0	0	0	0	X	X	H*	H
Spsp-UA*63	0	0	0	0	0	0	0	0	0	0	0	0
Spsp-UA*64	0	0	0	0	0	0	0	0	0	0	0	0
Spsp-UA*65	0	0	0	0	0	0	0	0	0	0	0	0
Spsp-UA*67	0	0	0	0	0	0	0	0	0	0	0	0
Spsp-UA*68	0	0	0	0	0	0	0	0	0	0	0	0
Spsp-UA*69	0	0	0	0	0	0	0	0	0	0	0	0
Spsp-UA*70	0	0	0	0	0	0	0	0	0	0	0	0
Spsp-UA*71	0	0	0	0	0	0	0	0	0	X	H	H
Spsp-UA*72	0	0	0	0	0	0	0	0	0	0	0	0
Spsp-UA*73	0	0	0	0	0	0	0	0	0	0	0	0
Spsp-UA*74	0	0	0	0	0	0	0	0	0	0	0	0
Spsp-UA*75	0	0	0	0	0	0	0	0	0	0	0	0
Spsp-UA*76	0	0	0	0	0	0	0	0	0	0	0	0
Spsp-UA*78	0	0	0	0	0	0	0	0	0	0	0	0
Spsp-UA*79	0	0	0	0	0	0	0	0	0	0	0	0
Spsp-UA*80	0	0	0	0	0	0	0	0	0	0	0	0
Spsp-UA*82	0	0	0	0	0	0	0	0	0	0	0	0
Spsp-UA*83	0	X	H	0	0	0	0	0	0	0	0	0
Spsp-UA*84	0	0	0	0	0	0	0	0	0	0	0	0
Spsp-UA*85	0	0	0	0	0	0	0	0	0	0	0	0
Spsp-UA*86	0	0	0	0	0	0	0	0	0	0	0	0
Spsp-UA*87	0	0	0	0	0	0	0	0	0	X	L	0
Spsp-UA*88	0	0	0	0	0	0	0	0	0	0	0	0
Spsp-UA*89	0	0	0	0	0	0	0	0	0	0	0	0
Spsp-UA*90	0	0	0	0	0	0	0	0	0	0	0	0
Spsp-UA*91	0	0	0	0	0	0	0	0	0	0	0	0

Allele	Individual 10				Individual 11				Individual 12			
	Primer combination 1 (gDNA)	Primer combination 2 (gDNA)	Primer combination 2 (cDNA)	Primer combination 3 (cDNA)	Primer combination 1 (gDNA)	Primer combination 2 (gDNA)	Primer combination 2 (cDNA)	Primer combination 3 (cDNA)	Primer combination 1 (gDNA)	Primer combination 2 (gDNA)	Primer combination 2 (cDNA)	Primer combination 3 (cDNA)
Spsp-UA*01	X	X	L	L	X	X	L	L	X	X	L	L
Spsp-UA*02	X	X	0	0	0	0	0	0	0	0	0	0
Spsp-UA*03	0	0	0	0	0	0	0	0	0	0	0	0
Spsp-UA*04	0	0	0	0	X	0	0	L	0	0	0	0
Spsp-UA*05	0	0	0	0	0	0	0	0	0	0	0	0
Spsp-UA*06	0	0	0	0	0	0	0	0	0	0	0	0
Spsp-UA*07	0	0	0	0	0	0	0	0	0	0	0	0
Spsp-UA*08	0	0	0	0	X	X	L	L	0	0	0	0
Spsp-UA*09	0	0	0	0	0	0	0	0	0	0	0	0
Spsp-UA*10	X	X	L	L	0	0	0	0	0	0	0	0
Spsp-UA*11	0	0	0	0	0	0	0	0	0	0	0	0
Spsp-UA*12	0	0	0	0	0	0	0	0	0	0	0	0
Spsp-UA*13	0	0	0	0	0	0	0	0	0	0	0	0
Spsp-UA*14	0	0	0	0	0	0	0	0	0	0	0	0
Spsp-UA*15	0	0	0	0	0	0	0	0	0	0	0	0
Spsp-UA*16	X	X	L	L	0	0	0	0	0	0	0	0
Spsp-UA*17	0	0	0	0	0	0	0	0	X	X	L	H
Spsp-UA*18	0	0	0	0	0	0	0	0	0	0	0	0
Spsp-UA*19	X	X	L	L	0	0	0	0	0	0	0	0
Spsp-UA*20	0	0	0	0	0	0	0	0	X	0	0	H
Spsp-UA*21	0	0	0	0	0	0	0	0	X	X	L	L
Spsp-UA*22	0	0	0	0	0	0	0	0	0	0	0	0
Spsp-UA*23	0	0	0	0	0	0	0	0	0	0	0	0
Spsp-UA*24	0	0	0	0	0	0	0	0	0	0	0	0
Spsp-UA*25	0	0	0	0	0	0	0	0	0	0	0	0
Spsp-UA*26	0	0	0	0	0	0	0	0	0	0	0	0
Spsp-UA*27	0	0	0	0	0	0	0	0	0	0	0	0
Spsp-UA*28	X	X	L	L	X	X	L	L	0	0	0	0
Spsp-UA*29	0	0	0	0	0	0	0	0	0	0	0	0
Spsp-UA*30	0	0	0	0	0	0	0	0	0	0	0	0
Spsp-UA*31	0	0	0	0	0	0	0	0	0	0	0	0
Spsp-UA*32	0	0	0	0	0	0	0	0	0	0	0	0
Spsp-UA*33	0	0	0	0	0	0	0	0	0	0	0	0
Spsp-UA*34	0	0	0	0	0	0	0	0	0	0	0	0
Spsp-UA*35	0	0	0	0	0	0	0	0	0	0	0	0
Spsp-UA*36	0	0	0	0	0	0	0	0	0	0	0	0
Spsp-UA*37	0	0	0	0	0	0	0	0	0	0	0	0
Spsp-UA*38	X	X	H	H	0	0	0	0	0	0	0	0
Spsp-UA*39	0	0	0	0	0	0	0	0	0	0	0	0
Spsp-UA*40	0	0	0	0	0	0	0	0	0	0	0	0
Spsp-UA*41	0	0	0	0	0	0	0	0	0	0	0	0
Spsp-UA*42	0	0	0	0	X	X	H*	H	0	0	0	0
Spsp-UA*43	0	0	0	0	0	0	0	0	0	0	0	0
Spsp-UA*44	0	0	0	0	0	0	0	0	0	0	0	0
Spsp-UA*45	0	0	0	0	0	0	0	0	0	0	0	0
Spsp-UA*46	0	0	0	0	0	0	0	0	0	0	0	0
Spsp-UA*47	0	0	0	0	0	0	0	0	0	0	0	0
Spsp-UA*48	0	0	0	0	0	0	0	0	X	0	0	L
Spsp-UA*49	0	0	0	0	0	0	0	0	0	0	0	0
Spsp-UA*50	0	0	0	0	0	0	0	0	0	0	0	0
Spsp-UA*51	0	0	0	0	X	X	L	L	0	0	0	0
Spsp-UA*52	0	0	0	0	0	0	0	0	0	0	0	0
Spsp-UA*53	0	0	0	0	0	0	0	0	0	0	0	0
Spsp-UA*54	0	0	0	0	0	0	0	0	0	0	0	0
Spsp-UA*55	0	0	0	0	0	0	0	0	0	0	0	0
Spsp-UA*56	0	0	0	0	0	0	0	0	0	0	0	0
Spsp-UA*57	0	0	0	0	0	0	0	0	0	0	0	0
Spsp-UA*58	0	X	H	H	0	0	0	0	0	0	0	0
Spsp-UA*59	0	0	0	0	0	0	0	L	0	X	H	H
Spsp-UA*60	0	0	0	0	0	X	H	H	0	0	0	0
Spsp-UA*61	0	0	0	0	0	X	H	H	0	0	0	0
Spsp-UA*62	0	0	0	0	0	0	0	0	0	0	0	0
Spsp-UA*63	0	0	0	0	0	0	0	0	0	X	H	H
Spsp-UA*64	0	0	0	0	0	0	0	0	0	0	0	0
Spsp-UA*65	0	0	0	0	0	0	0	0	0	0	0	0
Spsp-UA*67	0	0	0	0	0	0	0	0	0	0	0	0
Spsp-UA*68	0	0	0	0	0	0	0	0	0	X	H	0
Spsp-UA*69	0	0	0	0	0	0	0	0	0	0	0	0
Spsp-UA*70	0	0	0	0	0	0	0	0	0	0	0	0
Spsp-UA*71	0	0	0	0	0	0	0	0	0	0	0	0
Spsp-UA*72	0	0	0	0	0	0	0	0	0	0	0	0
Spsp-UA*73	0	0	0	0	0	0	0	0	0	0	0	0
Spsp-UA*74	0	0	0	0	0	0	0	0	0	0	0	0
Spsp-UA*75	0	0	0	0	0	0	0	0	0	0	0	0
Spsp-UA*76	0	0	0	H	0	0	0	0	0	0	0	0
Spsp-UA*78	0	0	0	0	0	0	0	0	0	0	0	0
Spsp-UA*79	0	0	0	0	0	0	0	0	0	0	0	0
Spsp-UA*80	0	X	H	0	0	0	0	0	0	0	0	0
Spsp-UA*82	0	0	0	0	0	0	0	0	0	0	0	0
Spsp-UA*83	0	0	0	0	0	0	0	0	0	0	0	0
Spsp-UA*84	0	0	0	0	0	0	0	0	0	0	0	0
Spsp-UA*85	0	0	0	0	0	0	0	0	0	0	0	0
Spsp-UA*86	0	0	0	0	0	0	0	0	0	0	0	0
Spsp-UA*87	0	0	0	0	0	X	L	0	0	0	0	0
Spsp-UA*88	0	0	0	0	0	0	0	0	0	0	0	0
Spsp-UA*89	0	0	0	0	0	0	0	0	0	0	0	0
Spsp-UA*90	0	0	0	0	0	0	0	0	0	0	0	0
Spsp-UA*91	0	0	0	0	0	0	0	0	0	0	0	0

Allele	Individual 13				Individual 14				Individual 16			
	Primer combination 1 (gDNA)	Primer combination 2 (gDNA)	Primer combination 2 (cDNA)	Primer combination 3 (cDNA)	Primer combination 1 (gDNA)	Primer combination 2 (gDNA)	Primer combination 2 (cDNA)	Primer combination 3 (cDNA)	Primer combination 1 (gDNA)	Primer combination 2 (gDNA)	Primer combination 2 (cDNA)	Primer combination 3 (cDNA)
Spsp-UA*01	X	X	L	L	X	X	L	L	X	X	L	L
Spsp-UA*02	0	0	0	0	0	0	0	0	0	0	0	0
Spsp-UA*03	0	0	0	0	0	0	0	0	0	0	0	0
Spsp-UA*04	X	0	0	L	X	0	0	L	X	0	0	L
Spsp-UA*05	X	0	0	L	0	0	0	0	0	0	0	0
Spsp-UA*06	0	0	0	0	0	0	0	0	0	0	0	L
Spsp-UA*07	X	X	H	H	0	0	0	0	0	0	0	0
Spsp-UA*08	0	0	0	0	0	0	0	0	0	0	0	0
Spsp-UA*09	X	X	L	L	0	0	0	0	0	0	0	0
Spsp-UA*10	0	X	L	0	0	0	0	0	0	0	0	0
Spsp-UA*11	0	0	0	0	0	0	0	0	0	0	0	0
Spsp-UA*12	0	0	0	0	0	0	0	0	0	0	0	0
Spsp-UA*13	0	0	0	0	X	X	L	L	0	0	0	0
Spsp-UA*14	0	0	0	0	0	0	0	0	0	0	0	0
Spsp-UA*15	0	0	0	0	0	0	0	0	0	0	0	0
Spsp-UA*16	0	0	0	0	0	0	0	0	X	X	L	L
Spsp-UA*17	0	0	0	0	0	0	0	0	0	0	0	0
Spsp-UA*18	0	0	0	0	X	0	0	L	0	0	0	0
Spsp-UA*19	0	0	0	0	0	0	0	0	0	0	0	0
Spsp-UA*20	0	0	0	0	0	0	0	0	0	0	0	0
Spsp-UA*21	0	0	0	0	0	0	0	0	0	0	0	0
Spsp-UA*22	0	0	0	0	X	X	L	L	0	0	L	0
Spsp-UA*23	0	0	0	0	X	X	H*	H	0	0	0	0
Spsp-UA*24	0	0	0	0	X	X	L	L	0	0	0	0
Spsp-UA*25	0	0	0	0	0	0	0	0	0	0	0	0
Spsp-UA*26	X	X	H	H	0	0	0	0	0	0	0	0
Spsp-UA*27	0	0	0	0	0	0	0	0	0	0	0	0
Spsp-UA*28	0	0	0	0	0	0	0	0	0	0	0	0
Spsp-UA*29	0	0	0	0	0	0	0	0	0	0	0	0
Spsp-UA*30	0	0	0	0	0	0	0	0	0	0	0	0
Spsp-UA*31	0	0	0	0	0	0	0	0	0	0	0	0
Spsp-UA*32	0	0	0	0	0	0	0	0	0	0	0	0
Spsp-UA*33	0	0	0	0	0	0	0	0	0	0	0	0
Spsp-UA*34	0	0	0	0	0	0	0	0	0	0	0	0
Spsp-UA*35	0	0	0	0	0	0	0	0	0	0	0	0
Spsp-UA*36	0	0	0	0	0	0	0	0	0	0	0	0
Spsp-UA*37	0	0	0	0	0	0	0	0	0	0	0	0
Spsp-UA*38	0	0	0	0	0	0	0	0	0	0	0	0
Spsp-UA*39	0	0	0	0	0	0	0	0	0	X	L	0
Spsp-UA*40	0	0	0	0	0	0	0	0	0	0	0	0
Spsp-UA*41	0	0	0	0	0	0	0	0	0	0	0	0
Spsp-UA*42	0	0	0	0	0	0	0	0	0	0	0	0
Spsp-UA*43	0	0	0	0	0	0	0	0	0	0	0	0
Spsp-UA*44	0	0	0	0	0	0	0	0	0	0	0	0
Spsp-UA*45	0	0	0	0	0	0	0	0	0	0	0	0
Spsp-UA*46	0	0	0	0	0	0	0	0	0	0	0	0
Spsp-UA*47	0	0	0	0	0	0	0	0	X	X	0	L
Spsp-UA*48	0	0	0	0	0	0	0	0	0	0	0	0
Spsp-UA*49	X	X	L	L	0	0	0	0	0	0	0	0
Spsp-UA*50	0	0	0	0	0	0	0	0	0	0	0	0
Spsp-UA*51	0	0	0	0	0	0	0	0	0	0	0	0
Spsp-UA*52	0	0	0	0	X	X	L	L	0	0	0	0
Spsp-UA*53	0	0	0	0	0	0	0	0	0	0	0	0
Spsp-UA*54	0	0	0	0	0	0	0	0	0	0	0	0
Spsp-UA*55	0	0	0	0	0	0	0	0	0	0	0	0
Spsp-UA*56	0	0	0	0	0	0	0	0	0	0	0	0
Spsp-UA*57	0	0	0	0	0	0	0	0	0	0	0	0
Spsp-UA*58	0	0	0	0	0	X	H	H	0	X	H	H
Spsp-UA*59	0	0	L	L	0	X	H	H	0	0	0	0
Spsp-UA*60	0	X	H	H	0	0	0	0	0	0	0	0
Spsp-UA*61	0	0	0	0	0	0	0	0	0	0	0	0
Spsp-UA*62	0	0	0	0	0	0	0	0	0	0	0	0
Spsp-UA*63	0	0	0	0	0	0	0	0	0	0	0	0
Spsp-UA*64	0	0	0	0	0	0	0	0	0	0	0	0
Spsp-UA*65	0	0	0	0	0	0	0	0	0	0	0	0
Spsp-UA*67	0	0	0	0	0	0	0	0	0	0	0	0
Spsp-UA*68	0	0	0	0	0	0	0	0	0	0	0	0
Spsp-UA*69	0	0	0	0	0	0	0	0	0	0	0	0
Spsp-UA*70	0	0	0	0	0	0	0	0	0	0	0	0
Spsp-UA*71	0	0	0	0	0	0	0	0	0	0	0	0
Spsp-UA*72	0	0	0	0	0	0	0	0	0	0	0	0
Spsp-UA*73	0	X	H	H	0	0	0	0	0	0	0	0
Spsp-UA*74	0	0	0	0	0	0	0	0	X	X	0	H
Spsp-UA*75	0	0	0	0	0	0	0	0	X	X	H	H
Spsp-UA*76	0	0	0	0	0	0	0	0	0	0	0	0
Spsp-UA*78	0	0	0	0	0	0	0	0	0	0	0	0
Spsp-UA*79	0	0	0	0	0	X	H	0	0	0	0	0
Spsp-UA*80	0	0	0	0	0	0	0	0	0	X	H	0
Spsp-UA*82	0	0	0	0	0	0	0	0	X	X	L	L
Spsp-UA*83	0	0	0	0	0	0	0	0	0	0	0	0
Spsp-UA*84	0	0	0	0	0	X	L	0	0	0	0	0
Spsp-UA*85	0	0	0	0	0	0	0	0	0	0	0	0
Spsp-UA*86	0	0	0	0	0	0	0	0	0	0	0	0
Spsp-UA*87	0	0	0	0	0	0	0	0	X	X	L	0
Spsp-UA*88	0	0	0	0	0	0	0	0	0	X	0	0
Spsp-UA*89	0	0	0	0	0	0	0	0	0	0	0	0
Spsp-UA*90	0	0	0	0	0	0	0	0	X	0	0	0
Spsp-UA*91	0	0	0	0	0	0	0	0	0	0	0	0

Allele	Individual 17 Primer combination 1 (gDNA)	Individual 17 Primer combination 2 (gDNA)	Individual 17 Primer combination 2 (cDNA)	Individual 17 Primer combination 3 (cDNA)	Individual 18 Primer combination 1 (gDNA)	Individual 18 Primer combination 2 (gDNA)	Individual 18 Primer combination 2 (cDNA)	Individual 18 Primer combination 3 (cDNA)	Individual 19 Primer combination 1 (gDNA)	Individual 19 Primer combination 2 (gDNA)	Individual 19 Primer combination 2 (cDNA)	Individual 19 Primer combination 3 (cDNA)
Spsp-UA*01	X	X	L	L	X	X	L	L	X	X	L	L
Spsp-UA*02	0	0	0	0	0	0	0	0	X	X	L	L
Spsp-UA*03	0	0	0	0	0	0	0	0	0	0	0	0
Spsp-UA*04	X	0	0	L	X	0	0	L	X	0	0	L
Spsp-UA*05	0	0	0	0	0	0	0	0	0	0	0	0
Spsp-UA*06	0	0	0	0	X	0	0	L	0	0	0	0
Spsp-UA*07	0	0	0	0	0	0	0	0	0	0	0	0
Spsp-UA*08	0	0	0	0	0	0	0	0	0	0	0	0
Spsp-UA*09	0	0	0	0	0	0	0	0	0	0	0	0
Spsp-UA*10	0	0	0	0	0	0	0	0	0	0	0	0
Spsp-UA*11	0	0	0	0	0	0	0	0	0	0	0	0
Spsp-UA*12	0	0	0	0	0	0	0	0	0	0	0	0
Spsp-UA*13	0	0	0	0	0	0	0	0	0	0	0	0
Spsp-UA*14	0	0	0	0	0	0	0	0	0	0	0	0
Spsp-UA*15	0	0	0	0	0	0	0	0	0	0	0	0
Spsp-UA*16	0	0	0	0	0	0	0	0	0	0	0	0
Spsp-UA*17	0	0	0	0	0	0	0	0	0	0	0	0
Spsp-UA*18	0	0	0	0	0	0	0	0	0	0	0	0
Spsp-UA*19	0	0	0	0	0	0	0	0	0	X	L	0
Spsp-UA*20	0	0	0	0	0	0	0	0	0	0	0	0
Spsp-UA*21	0	0	0	0	0	0	0	0	0	0	0	0
Spsp-UA*22	0	0	0	0	0	0	L	0	0	0	0	0
Spsp-UA*23	0	0	0	0	0	0	0	0	0	0	0	0
Spsp-UA*24	0	0	0	0	0	0	0	0	0	0	0	0
Spsp-UA*25	0	0	0	0	0	0	0	0	0	0	0	0
Spsp-UA*26	0	0	0	0	0	0	0	0	0	0	0	0
Spsp-UA*27	0	0	0	0	0	0	0	0	0	0	0	0
Spsp-UA*28	0	0	0	0	X	X	L	L	0	0	0	0
Spsp-UA*29	0	0	0	0	0	0	0	0	0	0	0	0
Spsp-UA*30	0	0	0	0	0	0	0	0	X	X	L	L
Spsp-UA*31	0	0	0	0	0	0	0	0	0	0	0	0
Spsp-UA*32	0	X	L	0	0	0	0	0	X	X	L	H
Spsp-UA*33	0	0	0	0	0	0	0	0	0	0	0	0
Spsp-UA*34	0	0	0	0	0	0	0	0	X	X	H	H
Spsp-UA*35	0	0	0	0	X	X	H	H	0	0	0	0
Spsp-UA*36	0	0	0	0	0	0	0	0	0	0	0	0
Spsp-UA*37	0	0	0	0	0	0	0	0	0	0	0	0
Spsp-UA*38	0	0	0	0	0	0	0	0	0	0	0	0
Spsp-UA*39	0	0	0	0	0	X	L	0	0	0	0	0
Spsp-UA*40	0	0	0	0	0	0	0	0	X	0	0	L
Spsp-UA*41	0	0	0	0	0	0	0	0	0	0	0	0
Spsp-UA*42	0	0	0	0	0	0	0	0	0	0	0	0
Spsp-UA*43	0	0	0	0	0	0	0	0	0	0	0	0
Spsp-UA*44	0	0	0	0	0	0	0	0	0	0	0	0
Spsp-UA*45	0	0	0	0	0	0	0	0	0	0	0	0
Spsp-UA*46	0	0	0	0	0	0	0	0	0	0	0	0
Spsp-UA*47	0	0	0	0	X	X	0	L	0	0	0	0
Spsp-UA*48	0	0	0	0	0	0	0	0	0	0	0	0
Spsp-UA*49	0	0	0	0	0	0	0	0	0	0	0	0
Spsp-UA*50	0	0	0	0	0	0	0	0	X	X	L	L
Spsp-UA*51	0	0	0	0	0	X	L	0	0	0	0	0
Spsp-UA*52	0	0	0	0	0	0	0	0	0	0	0	0
Spsp-UA*53	0	0	0	0	0	0	0	0	0	0	0	0
Spsp-UA*54	0	0	0	0	0	0	0	0	0	0	0	0
Spsp-UA*55	0	0	0	0	X	X	L	L	0	0	0	0
Spsp-UA*56	0	0	0	0	0	0	0	0	0	0	0	0
Spsp-UA*57	0	0	0	0	0	0	0	0	0	0	0	0
Spsp-UA*58	0	X	H	H	0	X	H	H	0	X	H	H
Spsp-UA*59	0	0	0	0	0	X	H	H	0	0	L	L
Spsp-UA*60	0	0	0	0	0	0	0	0	0	X	H	H
Spsp-UA*61	0	0	0	0	0	0	0	0	0	0	0	0
Spsp-UA*62	0	0	0	0	0	0	0	0	0	0	0	0
Spsp-UA*63	0	0	0	0	0	0	0	0	0	0	0	0
Spsp-UA*64	0	0	0	0	0	0	0	0	0	0	0	0
Spsp-UA*65	0	0	0	H	0	0	0	0	0	0	0	0
Spsp-UA*67	0	0	0	0	0	0	0	H	0	0	0	0
Spsp-UA*68	0	0	0	0	0	0	0	0	0	0	0	0
Spsp-UA*69	0	0	0	0	0	0	0	0	0	0	0	0
Spsp-UA*70	X	0	0	L	0	0	0	0	0	0	0	0
Spsp-UA*71	0	0	0	0	0	0	0	0	0	0	0	0
Spsp-UA*72	0	0	0	0	0	0	0	0	0	0	0	0
Spsp-UA*73	0	0	0	0	0	0	0	0	0	0	0	0
Spsp-UA*74	0	0	0	0	0	0	0	0	0	0	0	0
Spsp-UA*75	0	0	0	0	0	0	0	0	0	0	0	0
Spsp-UA*76	0	0	0	0	0	0	0	0	0	0	0	0
Spsp-UA*78	0	0	0	0	0	0	0	0	0	0	0	0
Spsp-UA*79	0	0	0	0	0	0	0	0	0	0	0	0
Spsp-UA*80	0	0	0	0	0	0	0	0	0	0	0	0
Spsp-UA*82	0	0	0	0	0	0	0	0	0	0	0	0
Spsp-UA*83	0	0	0	0	0	0	0	0	0	0	0	0
Spsp-UA*84	0	0	0	0	0	0	0	0	0	0	0	0
Spsp-UA*85	X	X	L	L	0	0	0	0	0	0	0	0
Spsp-UA*86	0	0	0	0	0	X	L	0	0	0	0	0
Spsp-UA*87	0	0	0	0	0	0	0	0	0	0	0	0
Spsp-UA*88	0	0	0	0	0	0	0	0	0	0	0	0
Spsp-UA*89	0	0	0	0	0	0	0	0	0	0	0	0
Spsp-UA*90	0	0	0	0	0	0	0	0	0	0	0	0
Spsp-UA*91	0	0	0	0	0	0	0	0	0	0	0	0

Supplementary Table 2: There was a discrepancy between the number of non-classical (N=13) and classical (N=54) MHC-I exon 3 siskin alleles and in order determine how this affected how many sites that were indicated as positively selected by the CODEML analysis we also run a random subset of 13 classical alleles which was repeated 10 times, each time on a new subset of 13 randomly picked classical alleles. When the analysis was run on all classical alleles six sites were identified as positively selected (position 1, 4, 6, 41, 45 and 53 of the alignment in Figure 5) whereas between two and five sites were identified in the repeated runs on 13 randomly selected classical alleles. The positions of the sites in the repeated runs completely corresponds to those in the identified when the analysis was run on all classical alleles.

Run	Amino acid position					
	1	4	6	41	45	53
1			X	X	X	X
2	X	X			X	X
3		X	X		X	X
4			X		X	
5	X	X	X		X	X
6	X				X	
7		X			X	X
8	X	X	X		X	X
9	X	X	X		X	X
10	X	X			X	X

Supplementary Table 3: a) Five different primer combinations were used for the initial amplification of exon 2-4 in one siskin individual. All primers had previously been designed to amplify MHC-I in passerines. The primer combinations included two previously unpublished primers (SongF2 and SongR3), HNalla which was designed to amplify MHC-I in great read warbler but amplifies satisfactory also in other passerine species (Westerdahl et al., 2004), FWD3 and RVS3b which were designed to amplify MHC-I across the family of Passerida (O'Connor et al., 2016) and Rv3 which was designed to amplify MHC-I in house sparrow (Karlsson and Westerdahl, 2013). b) Four new primers were designed that amplified exon 3 in siskins and in total tree primer combinations, based on these new primers as well as HNalla, was used to amplify MHC-I exon 3 with illumina amplicon sequencing.

a)

Primer combination	Forward primer name	Forward primer sequences	Reverse primer name	Reverse primer sequences	Fragment length (bp)	Annealing temperature
1	SongF2	5' CRCAGTTCTCCACTCCCTGC	SongR3	5' ATCCCGGGCTCCRGCATTCCT	766	66°C
2	HNalla	5' TCCCCACAGGTCTCCACAC	SongR3		502	64°C
3	FWD3	5' TGGTTGCGAGTTTACGGYTRTG	SongR3		482	60°C
4	SongF2		RVS3b	5' TGGTTGCGAGTTTACGGYTRTG	449	60°C
5	SongF2		Rv3	5' TGCGCTCCAGCTCCYTCTGCC	501	64°C

b)

Primer combination	Forward primer name	Forward primer sequences	Reverse primer name	Reverse primer sequences	Amplicon size (bp)	Annealing temperature
1	HNalla	5' TCCCCACAGGTCTCCACAC	Spsps_rvs1	5' CCGACGTATTTCCRGAGCC	215	66°C
2	Spsps_fwd1	5' CCTCCTGTCTGATGSGAGTGTC	Spsps_rvs2	5' TTGCGCTCCAGCTCCYTCT	196	66°C
3	Spsps_fwd3	5' CARGARCGRTACAACCAGAGC	Spsps_rvs1	5' CCGACGTATTTCCRGAGCC	229	66°C

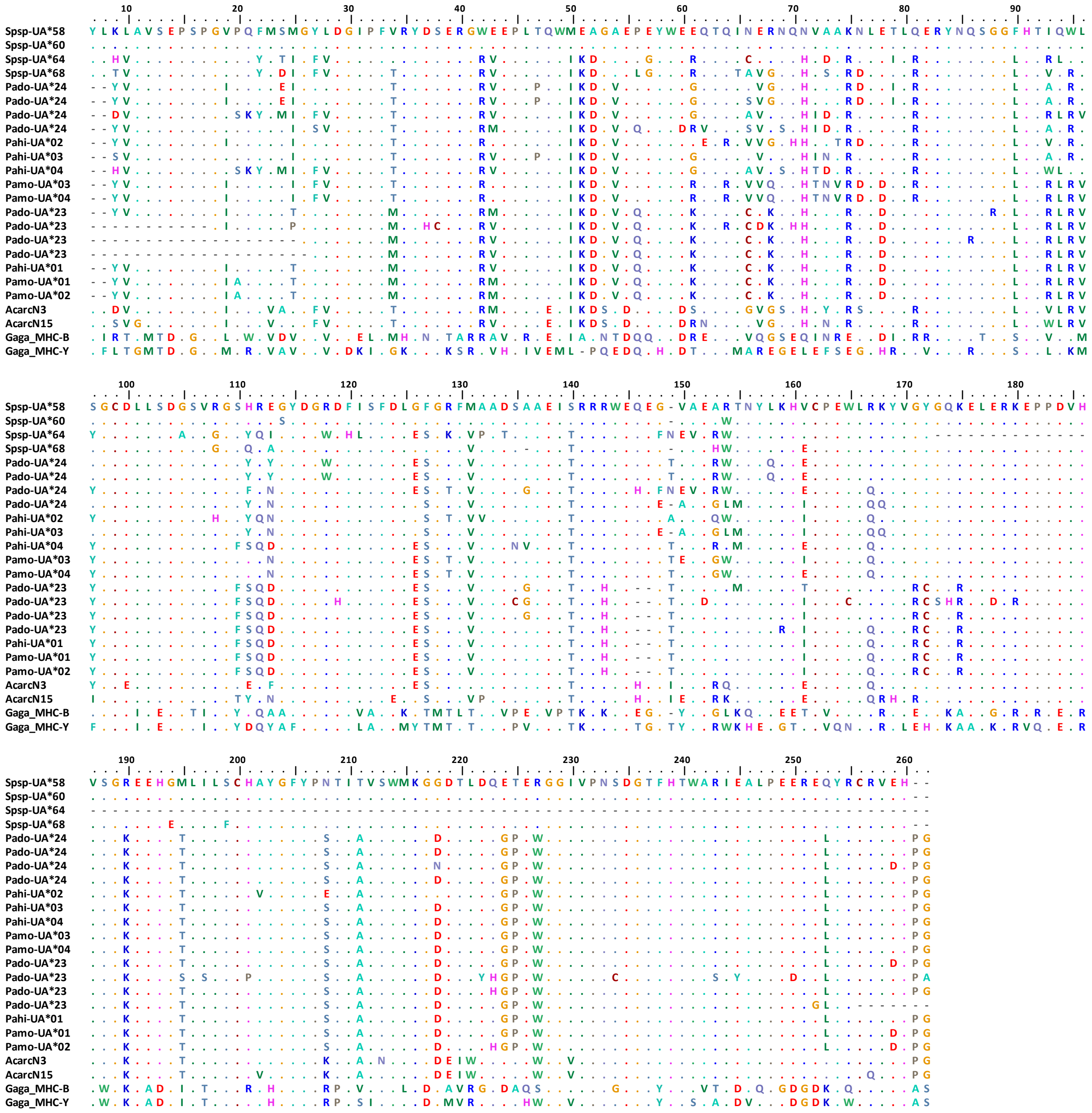

Supplementary Fig. 1: Amino acid alignment of passerine bird MHC-I exon 2-4, displaying four new verified siskin MHC-I alleles (Spsp) where the name corresponds to the Miseq exon 3 alleles, eight house sparrow alleles (Pado), four Spanish sparrow alleles (Pahi), four tree sparrow alleles (Pamo), two great read warbler alleles (Acar, GenBank acc nr: AJ005503.1 and AJ005505.1) and two chicken alleles (Gaga, GenBank acc nr: HQ141386.1 and NM_001030675.2). Dots represents similarity to Spsp-UA*58, and Pado-UA*230 to Pado-UA*233, Pahi-UA*01, Pamo-UA*01 and Pamo-UA*02 are putatively non-classical alleles as is Gaga_MHC-Y.

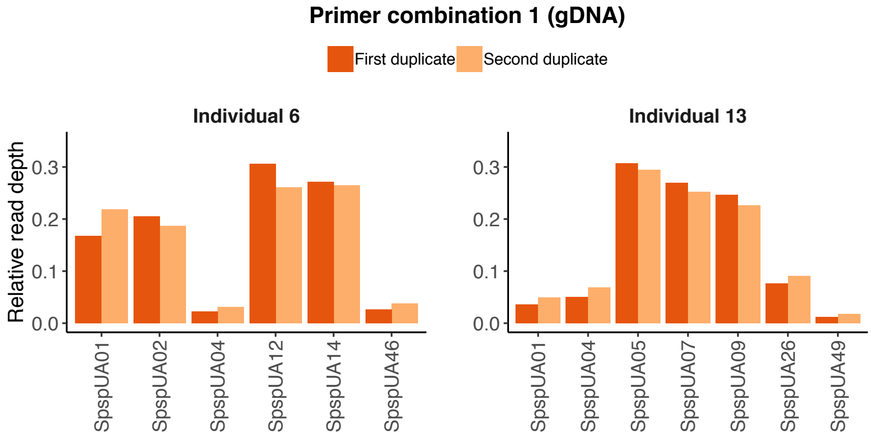
Primer combination 1 (gDNA)
First duplicate
Second duplicate
Individual 6
Individual 13
Relative read depth
0.0
0.1
0.2
0.3
SpspUA01
SpspUA02
SpspUA04
SpspUA12
SpspUA14
SpspUA46
SpspUA01
SpspUA04
SpspUA05
SpspUA07
SpspUA09
SpspUA26
SpspUA49

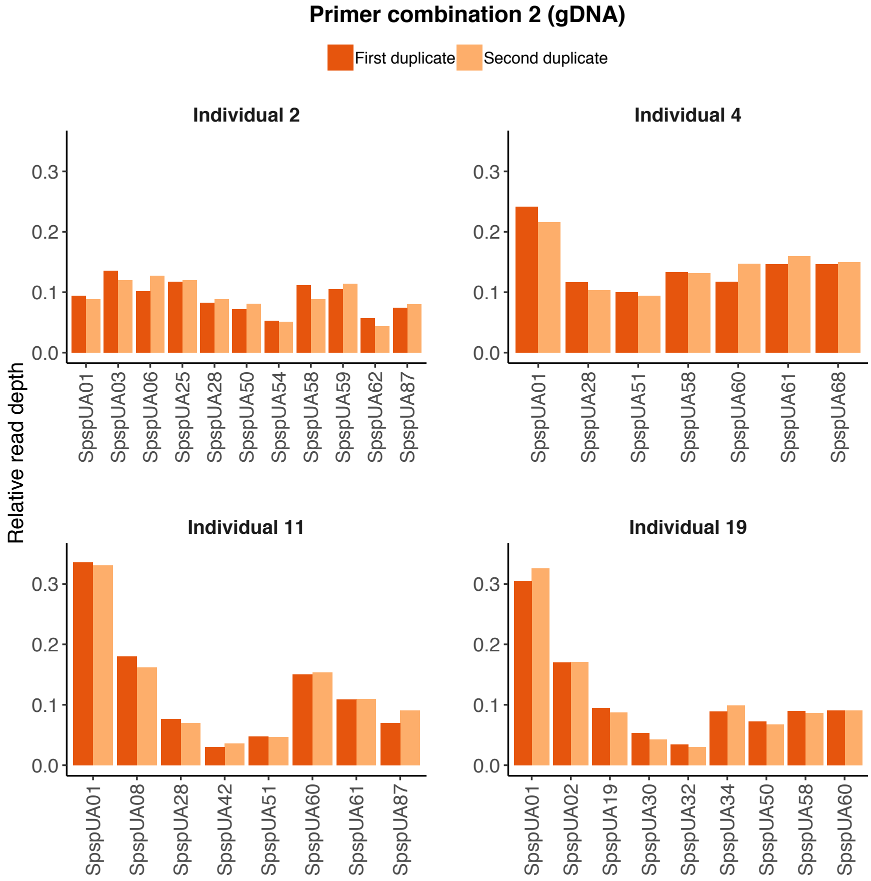
Primer combination 2 (gDNA)
First duplicate
Second duplicate
Individual 2
Individual 4
Individual 11
Individual 19
Relative read depth
0.0
0.1
0.2
0.3
SpspUA01
SpspUA03
SpspUA06
SpspUA25
SpspUA28
SpspUA50
SpspUA54
SpspUA58
SpspUA59
SpspUA62
SpspUA87
SpspUA01
SpspUA28
SpspUA51
SpspUA58
SpspUA60
SpspUA61
SpspUA68
SpspUA01
SpspUA08
SpspUA28
SpspUA42
SpspUA51
SpspUA60
SpspUA61
SpspUA87
SpspUA01
SpspUA02
SpspUA19
SpspUA30
SpspUA32
SpspUA34
SpspUA50
SpspUA58
SpspUA60

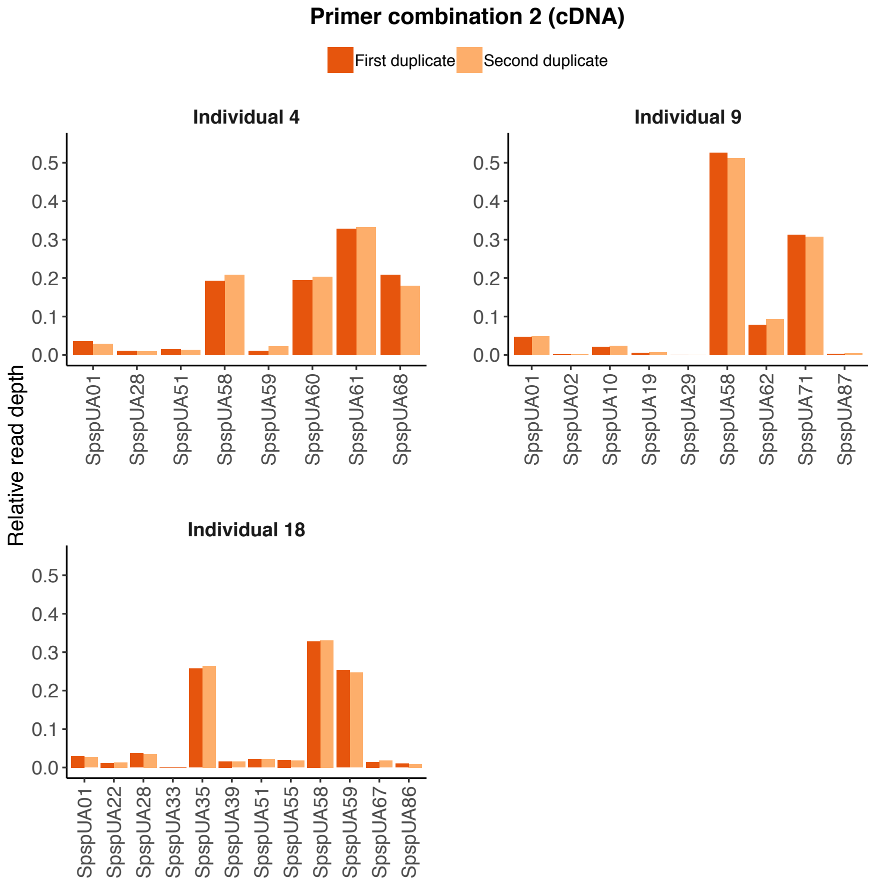

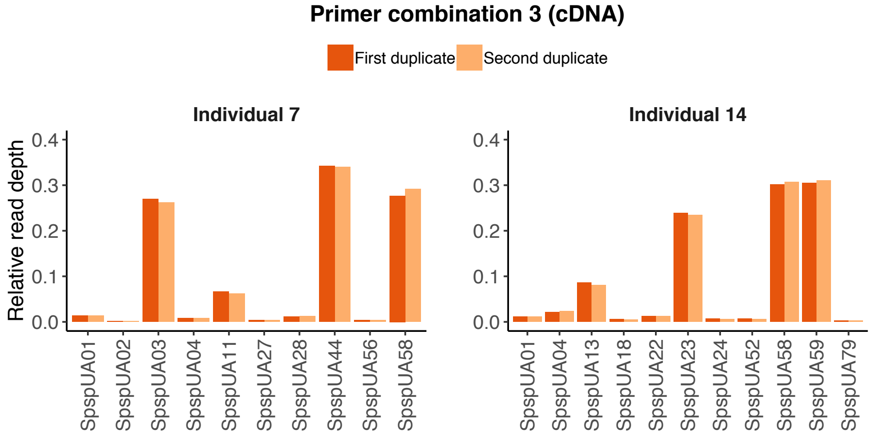

Supplementary Fig. 2: In order to estimate the repeatability and reliability of the Illumina sequencing of MHC-I exon 3 in siskins the duplicates were compared. Four duplicates were included for the gDNA samples amplified with primer combinations 1 and 2, respectively, three duplicates were included for the cDNA samples amplified with primer combinations 2 and 3, respectively. For primer combination 1 two of the duplicates failed and no sequences were retrieved, for primer combination 3 one duplicate failed. For all duplicates, independent of primer combination and DNA type (gDNA/cDNA), the same alleles were identified and the relative read depth per allele was also highly similar when comparing the duplicates.

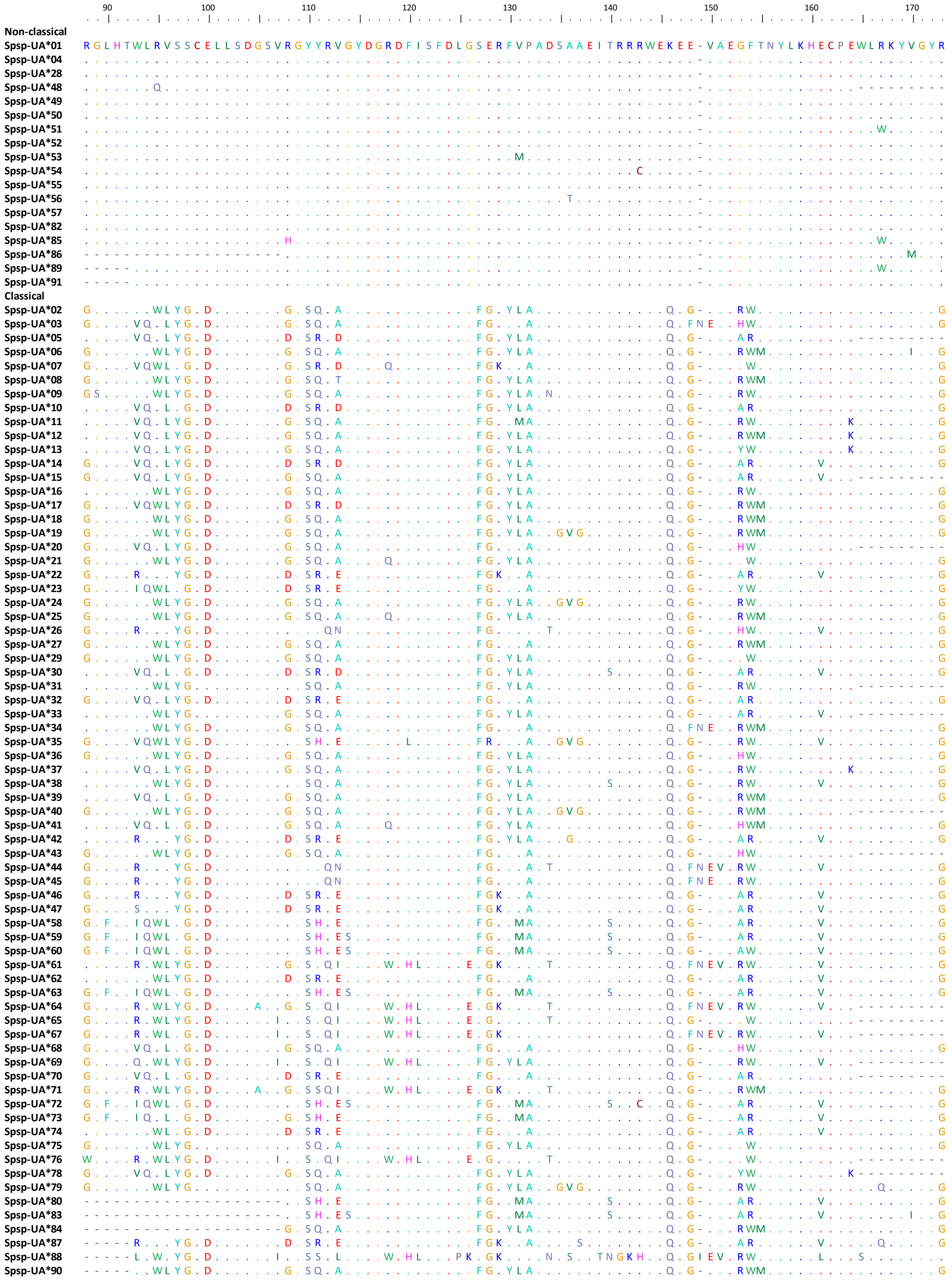

Supplementary Fig. 3: Amino acid alignment of Miseq exon 3 alleles from siskin, dots represent similarities to allele Spsp*UA01. Alleles have been amplified with three different primer combinations (PP1-PP3), the 18 non-classical alleles are Spsp-UA*01, 04, 48-57, 82, 85, 86, 89 and 91. The alleles Spsp-UA*01, 04, 28, 49, 50, 52, 55, 57, 82 are all identical in the amino acid sequence but are different in the nucleotide sequence, as are Spsp-UA*18 and 84, Spsp-UA*58 and 80, Spsp-UA*59 and 65.

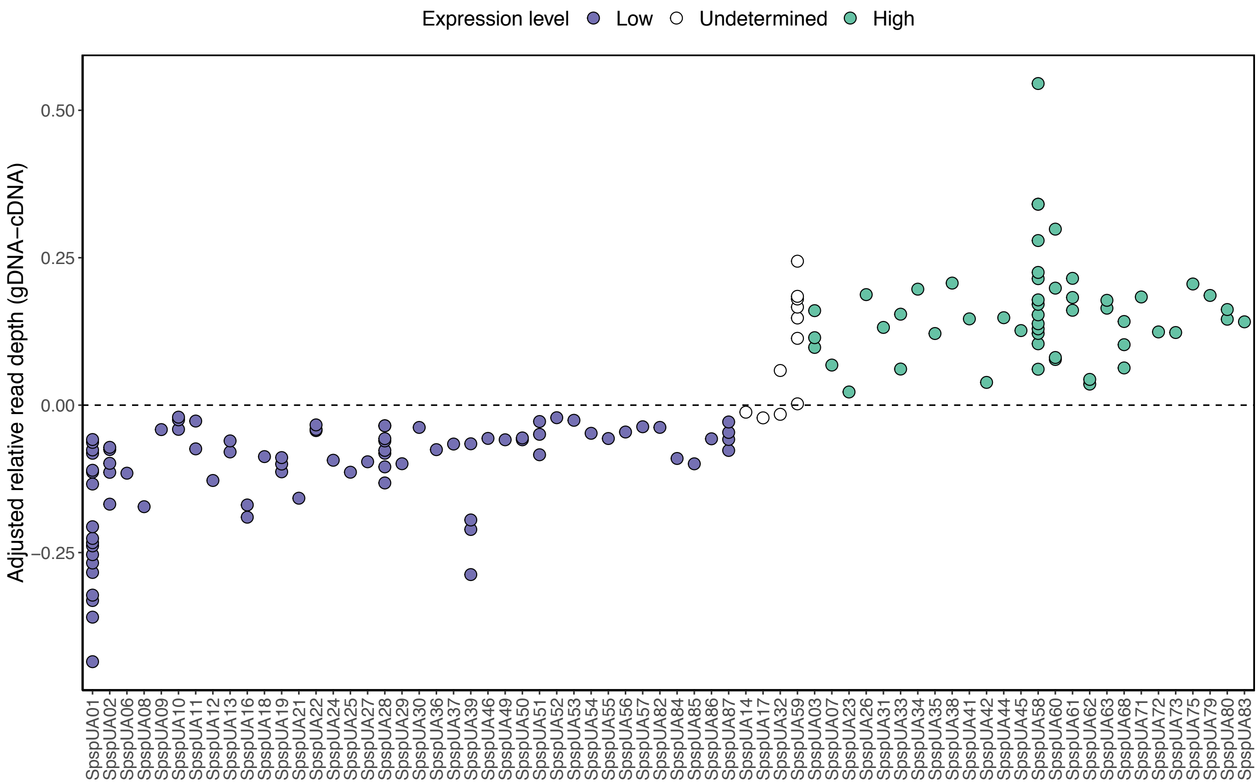

Supplementary Fig. 4: Primer combination 2 was used to amplify both gDNA and cDNA alleles, and for the alleles that were amplified with this primer combination (the 68 expressed siskin MHC-I alleles that are illustrated in this figure) the degree of expression could be determined by comparing the relative read depths per allele in gDNA and cDNA. This adjusted relative read depth was calculated as relative read depth in gDNA minus relative read depth in cDNA. A low expression allele (purple) should have an adjusted read depth below zero and a high expression allele (green) should have an adjusted read depth higher than zero. The degree of expression could not be determined for four alleles (white, see Methods section for further details). In the full data set (not shown in this figure) 80 out of the 84 expressed siskin MHC-I alleles were divided into the high or low expression groups depending on their relative read depths in cDNA. The division of alleles into these groups were determined, when possible, by combining information from both primer combination 2 and 3 (primers used when amplifying cDNA). Fifty-nine of the 80 expressed alleles were amplified with both primer combination 2 and 3, six alleles were only amplified with primer combination 2 and 16 alleles were only amplified with primer combinations 3. All alleles that had an adjusted relative read depth below zero (indicated in purple in this figure) were also determined as having low expression when using the full data set and information from primer combinations 2 and 3. Likewise all alleles that had an adjusted relative read depth above zero (indicated in green in this figure) were also determined as having high expression when using the full data set and information from primer combinations 2 and 3. Hence, the two methods used to determine degree of expression level agreed to 100%.

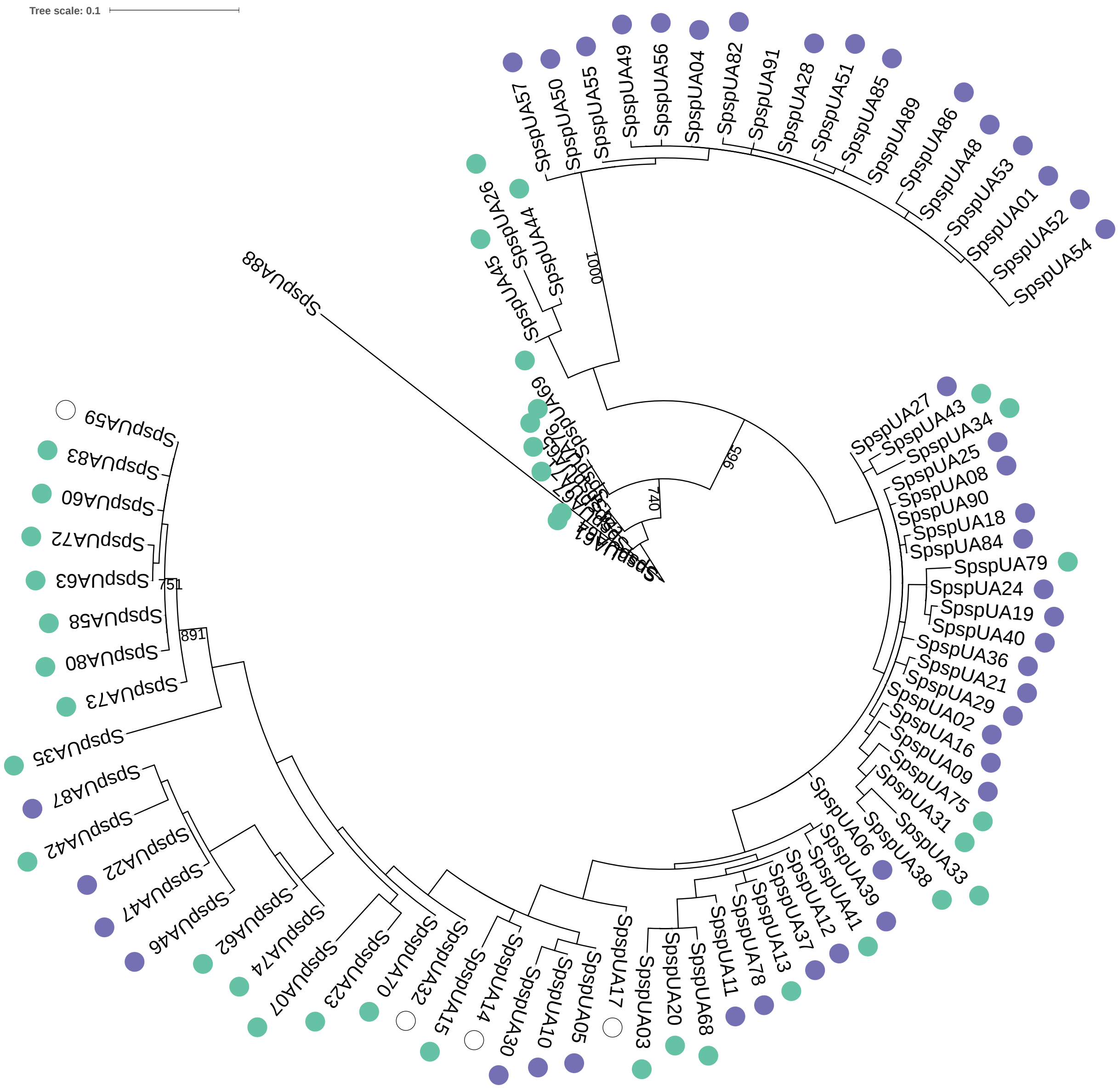

Supplementary Fig. 5: Maximum likelihood tree based on 88 MHC class I exon 3 nucleotide sequences from siskins. The tree was constructed with PhyML software (version 3.1.2) using the K80 model with gamma distribution and 1000 bootstraps, displaying bootstraps values higher than 700. Green circles represent alleles with high expression, purple circles represent alleles with low expression, white circles represent alleles with undetermined expression and alleles without circles are not expressed. The 18 putatively non-classical are found in one highly supported cluster (bootstrap 1000). Branch length indicates the divergence between alleles.

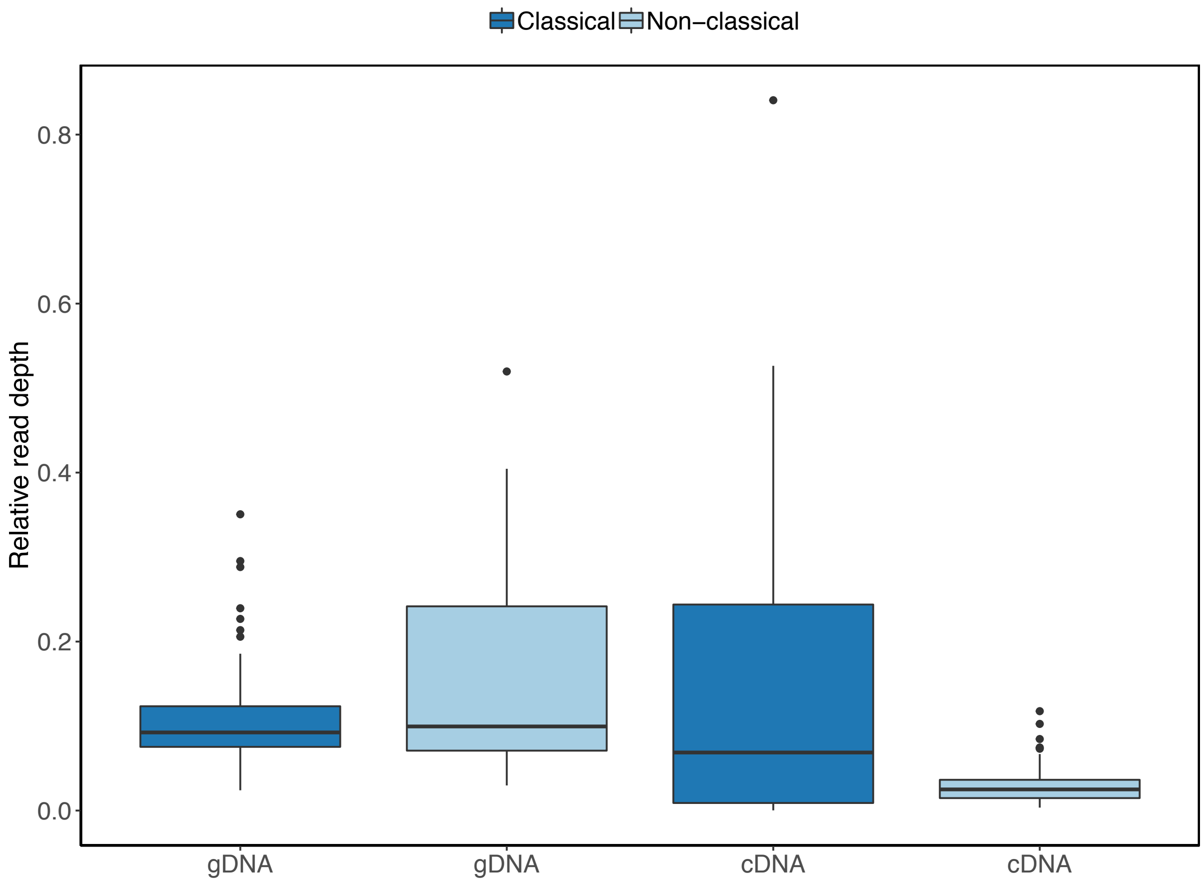

Supplementary Fig. 6: Box plot displaying variance in relative read depth per allele in each of 18 siskin individuals when comparing classical (dark blue) and non-classical alleles (light blue) that have been amplified by primer combination 2 in gDNA and cDNA samples respectively. The number of alleles varies between the groups; classical: gDNA N=110, cDNA N=117, non-classical: gDNA N=41, cDNA=41.

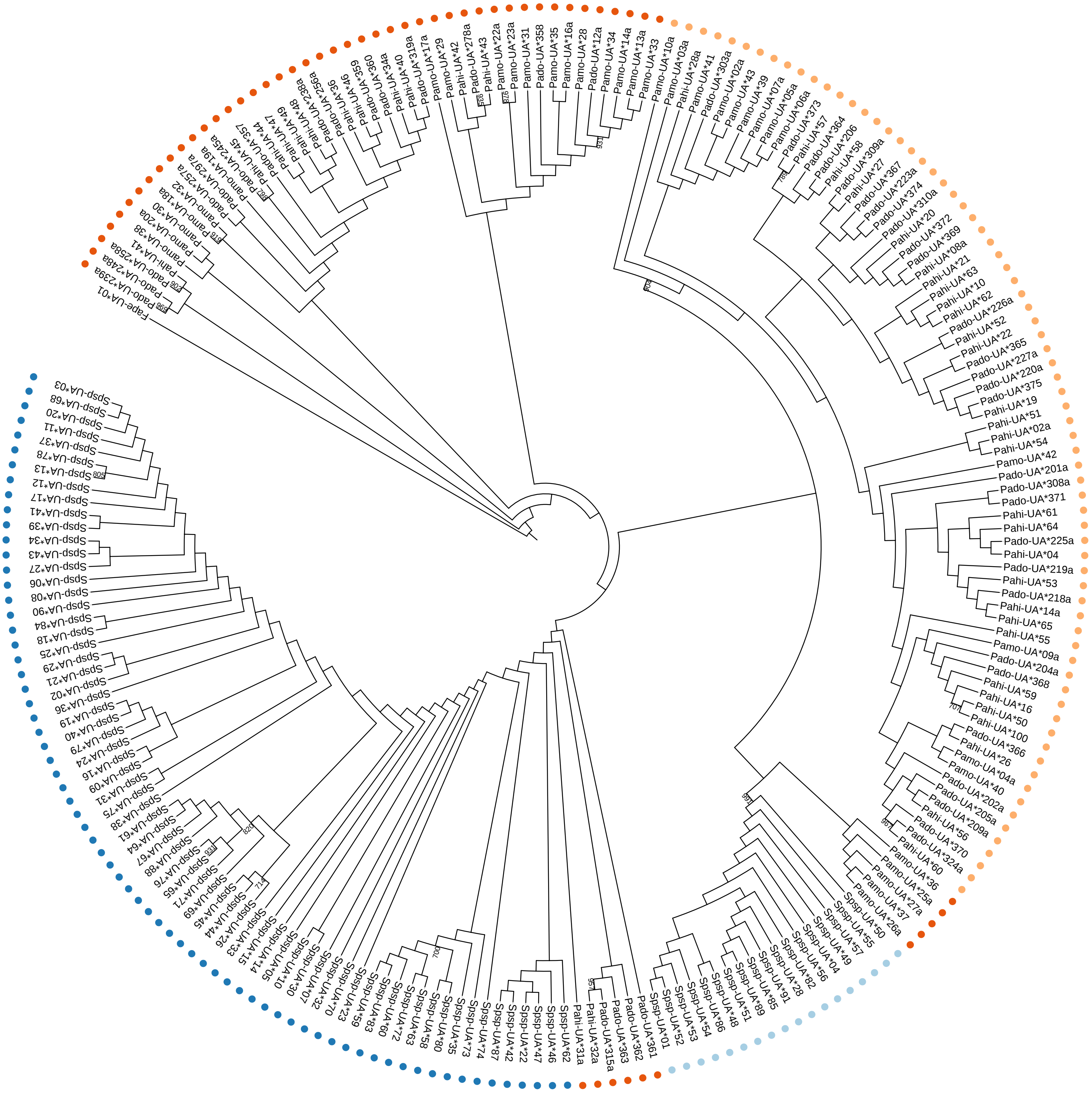

Supplementary Fig. 7: Maximum likelihood tree based on MHC class I exon 3 nucleotide sequences from siskins, house sparrows, tree sparrows and Spanish sparrows. One MHC class I sequence (Acc nr JN613264) from *Falco peregrinus* was used as the outgroup. The tree was constructed with PhyML software (version 3.1.2) using the HKY model with gamma distribution and 1000 bootstraps, displaying bootstraps values higher than 700. Dark blue circles represent the 70 classical alleles identified in siskins, light blue circles represent the 18 non-classical alleles identified in siskins, dark orange circles represent the 56 classical alleles identified in the sparrows by Drews et al. 2017 and light orange circles represent the 73 non-classical alleles identified in sparrows. The non-classical alleles from all three sparrow species are found in one clade and the non-classical alleles from siskins are found in another separate cluster.

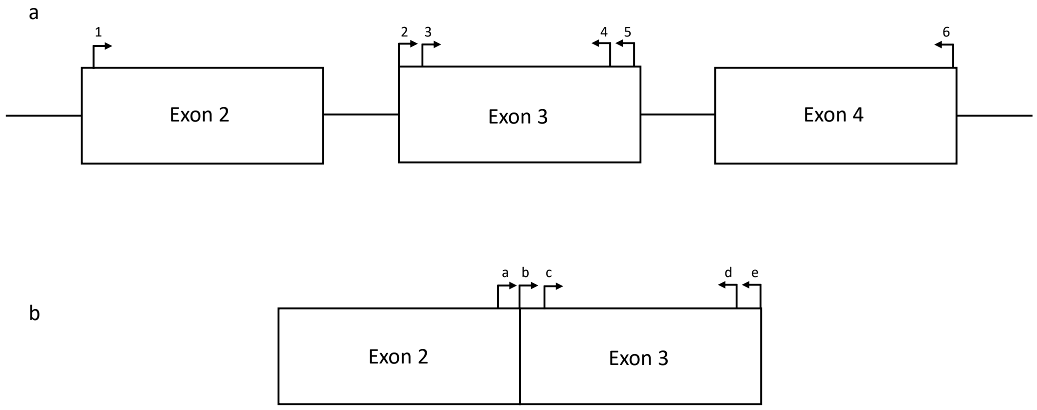

Supplementary Fig. 8: Primers used for MHC amplification in siskins, arrows indicates positions of primers. a) Primers used for initial amplification of exon 2-4 using six different primers (1-SongF2, 2-HNalla, 3-FWD3, 4-RVS3b, 5-Rv3, 6-SongR3) in total of five combinations (Primer combinations: 1+6, SongF2 – SongR3, 2+6, HNalla – Song3R, 3+6, FWD3 – SongR3, 1+4, SongF2 – RVS3b, 1+5, SongF2 – Rv3). b) Primers used for amplification of exon 3 and Illumina MiSeq using five different primer (a-Spsps_fwd3, b-HNalla, c-Spsps_fwd1, d-Spsps_rvs1, e-Spsps_rvs2) in total of three combinations (primer combination 1, b+d, HNalla – Spsps_rvs1, primer combination 2, c+e, Spsps_fwd1 – Spsps_rvs2, primer combination 3, a+d, Spsps_fwd3 – Spsps_rvs1).

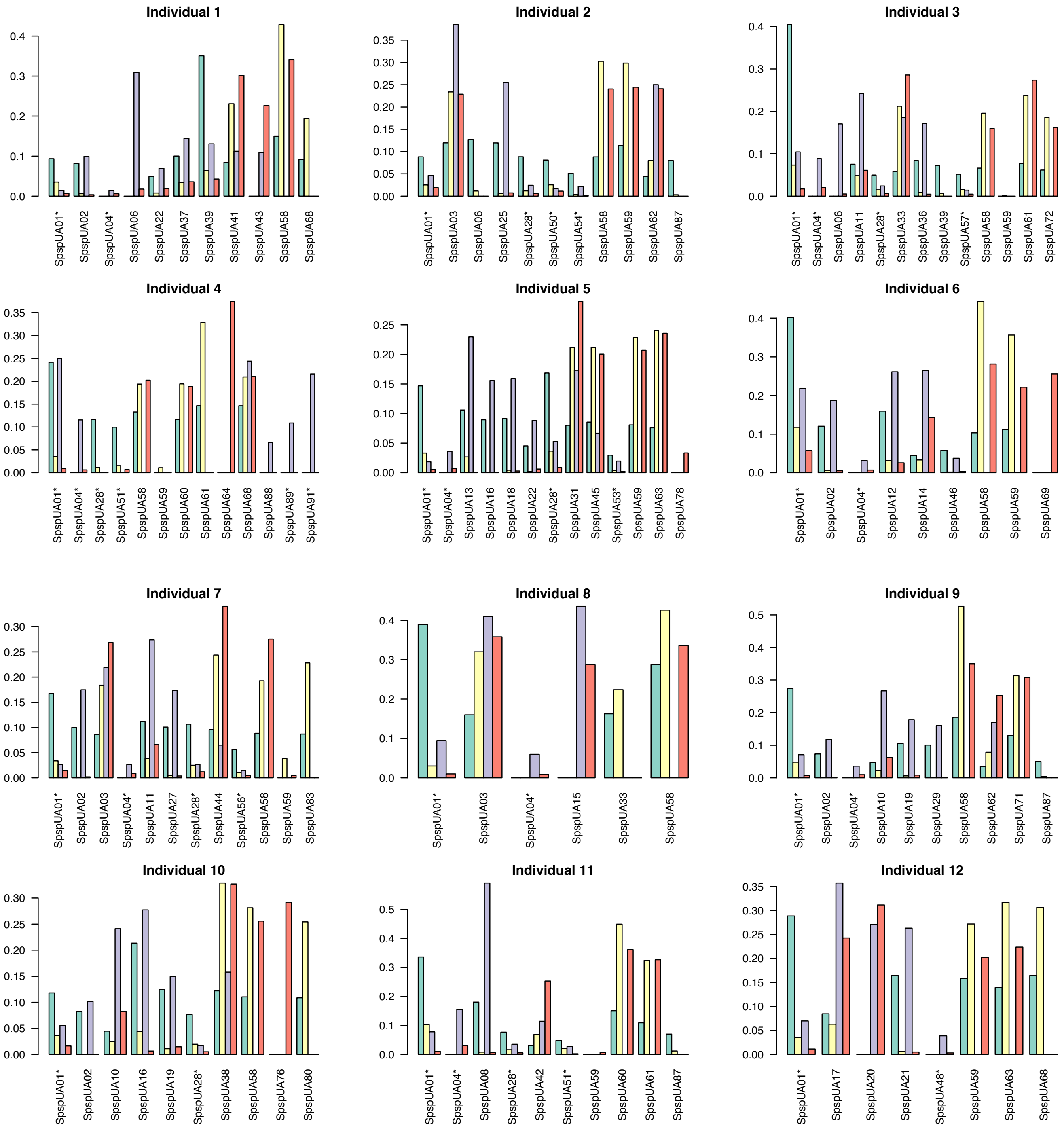
Individual 1
SpspUA01* SpspUA02 SpspUA04* SpspUA06 SpspUA22 SpspUA37 SpspUA39 SpspUA41 SpspUA43 SpspUA58 SpspUA68
Individual 2
SpspUA01* SpspUA03 SpspUA06 SpspUA25 SpspUA28* SpspUA50* SpspUA54* SpspUA58 SpspUA59 SpspUA62 SpspUA87
Individual 3
SpspUA01* SpspUA04* SpspUA06 SpspUA11 SpspUA28* SpspUA33 SpspUA36 SpspUA39 SpspUA57* SpspUA58 SpspUA59 SpspUA61 SpspUA72
Individual 4
SpspUA01* SpspUA04* SpspUA28* SpspUA51* SpspUA58 SpspUA59 SpspUA60 SpspUA61 SpspUA64 SpspUA68 SpspUA88 SpspUA89* SpspUA91*
Individual 5
SpspUA01* SpspUA04* SpspUA13 SpspUA16 SpspUA18 SpspUA22 SpspUA28* SpspUA31 SpspUA45 SpspUA53* SpspUA59 SpspUA63 SpspUA78
Individual 6
SpspUA01* SpspUA02 SpspUA04* SpspUA12 SpspUA14 SpspUA46 SpspUA58 SpspUA59 SpspUA69
Individual 7
SpspUA01* SpspUA02 SpspUA03 SpspUA04* SpspUA11 SpspUA27 SpspUA28* SpspUA44 SpspUA56* SpspUA58 SpspUA59 SpspUA83
Individual 8
SpspUA01* SpspUA03 SpspUA04* SpspUA15 SpspUA33 SpspUA58
Individual 9
SpspUA01* SpspUA02 SpspUA04* SpspUA10 SpspUA19 SpspUA29 SpspUA58 SpspUA62 SpspUA71 SpspUA87
Individual 10
SpspUA01* SpspUA02 SpspUA10 SpspUA16 SpspUA19 SpspUA28* SpspUA38 SpspUA58 SpspUA76 SpspUA80
Individual 11
SpspUA01* SpspUA04* SpspUA08 SpspUA28* SpspUA42 SpspUA51* SpspUA59 SpspUA60 SpspUA61 SpspUA87
Individual 12
SpspUA01* SpspUA17 SpspUA20 SpspUA21 SpspUA48* SpspUA59 SpspUA63 SpspUA68

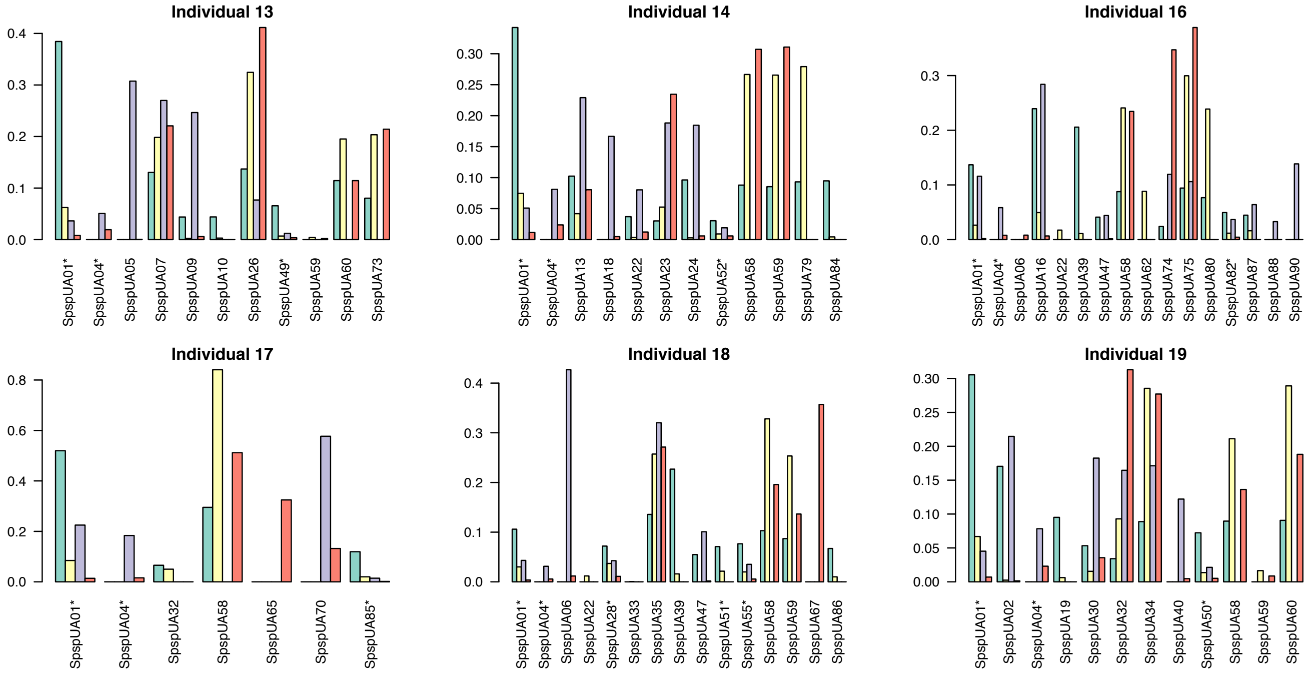

Supplementary Fig. 9: Relative read depth of MHC-I exon 3 alleles, displayed for all 18 siskin individuals. Green bars are alleles amplified by primer combination 2 in gDNA, yellow bars are alleles amplified by primer combination 2 in cDNA, purple bars are alleles amplified by primer combination 1 in gDNA and pink bars are alleles amplified by primer combination 3 in cDNA, stars (*) indicates non-classical alleles, numbers above plots correspond to siskin individual 1-18.

Supplementary methods

Sequencing of exon 2-4 and primer design

Since no MHC-I sequences from siskin have been previously published we used five different primer combinations from other songbird species to initially amplify MHC-I exon 2-4 in one siskin individual. All PCR's were run using 10 ng of cDNA as template with standard PCR protocols (94°C-30s, X°C-30s, 72°C-1:30min for 30 cycles) using AmpliTaq polymerase kit (Applied Biosystems), for annealing temperatures see Supplementary Table 1. The PCR products were checked on 2% agarose gels and PCR products of the correct length were then cloned using the TOPO TA Cloning Kit with the pCR2.1 TOPO vector and One Shot chemically competent cells, following manufacturers protocol (Invitrogen). Positive colonies (white) were transferred to tubes containing 150 µl of double-distilled water and then heated to 95°C for 3 min in order to lyse the bacteria. After which a new PCR was run using 1-2 µl of lysed bacteria as template and with the forward primer M13F and the reverse primer M13R, (Invitrogen). The PCR products were run on 2% agarose gels in order to determine which bacterial clones that had an insert of correct length. Successful PCR samples were then used as template in a BigDye terminator sequencing reaction using BigDye terminator kit v.3.1. Sequences were obtained from an ABI PRISM 3130 genetic analyzer (Applied Biosystems) at the DNA sequencing facility, Department of Biology, Faculty of Science, Lund University.

Illumina MiSeq sequencing of exon 3

We tested five different primer combinations that amplify MHC-I exon 3 alleles and out of these three primer combinations showed good results and were used for the Illumina sequencing (Supplementary Fig. 4). The binding site of HNalla is partly located in introns between exon 2 and exon 3 and hence it was only used on gDNA whereas Spsps_fwd3 is located in the end of exon 2 and because of this it was only used on cDNA. To each of these

primers the Illumina-specific overhang was added before ordering the primers and then used to amplify MHC-I exon 3. Each 25 µl PCR reaction contained either 25 ng gDNA or 25 ng cDNA, 12.5 µl 2X Phusion High-Fidelity PCR Master Mix (ThermoFisher Scientific), 0.5 µM of each primer. The cycling conditions for all three primer combinations were set to 25 cycles at 98°C (10s), 66°C (10s), 72°C (10s) followed by 72°C for ten minutes.

The PCR products were cleaned with Agencourt AMPure XP-PCR Purification kit (Beckman Coulter), following the manufacturer's protocol with slight modifications. A ratio of 1:0.8 between PCR product and beads was used, 80% EtOH was used for cleaning the beads and the elution was done with 43 µl double distillated water and incubation was done in room temperature for 2 minutes. The cleaned PCR products were then checked on a 2% agarose gel in order to determine that fragments of the correct length had been amplified, and the concentrations of the samples were estimated based on the strength of the bands.

To allow recovery of individual amplicons after demultiplexing, unique combinations of forward and reverse Illumina indexes were added to each sample by using Nextera XT v2 Index Kit (Illumina Inc., San Diego, CA, USA). Each 50 µl PCR reaction contained 25 µl 2X Phusion High-Fidelity PCR Master Mix (ThermoFisher Scientific), 5 µl of each index primers and 5,10 or 15 µl of cleaned PCR amplicon product, depending on the estimated concentration. The PCR profile was set to eight cycles at 98°C (10 s), 62°C (15 s) and 72°C (15 s), ending with 72°C for 10 minutes.

The indexed PCR products were cleaned with Agencourt AMPure XP-PCR Purification kit (Beckman Coulter), following the manufacturer's protocol with slight modifications. A ratio of 1:1.12 between PCR product and beads was used, 80% EtOH was used for cleaning,

elution was done with 43 µl double distillated water and incubation was carried out in room temperature for 2 minutes. The result was again checked on a 2% agarose gel and the concentration of each sample was measured with Quant-iT PicoGreen dsDNA Assay Kit (ThermoFisher Scientific) modified for a 96 well plate and measured on a plate reader.

Equimolar quantities of every sample were pooled into five pools depending on concentration and primer combination. These pools were then quantified with Qubit (ThermoFisher) and run on a Bioanalyzer DNA 2100 chip for quality and size validation. Lastly, equimolar quantities of all five pools were combined to a 4 nM library. The final library was sent for 300 bp paired-end Illumina MiSeq sequencing at the DNA sequencing facility, Department of Biology, Faculty of Science, Lund University.

Filtering of Illumina Miseq data

The primers were removed from all reads with cutadapt v 1.14[2]. In order to make sure that the primers had been properly removed from all reads we searched for the primer sequences and found that it still remained in some of the reads and these reads were also longer than expected. In order to remove the primer sequences and the extra bases all reads where cut to the expected fragment length for each primer combination (primer combination 1: 215bp, primer combination 2:196, primer combination 3: 229). Then we searched for the primer sequences again which were not found. To determine the quality of the sample and evaluate the samples we used FastQC v 0.11.7[3] and MultiQC v 1.4[4]. Overall, all the majority of the raw reads had good quality with an average phred score above 30, although primer combination 3 had slightly lower quality. To further process the raw reads we used DADA2, a program that can remove reads that have too low quality, by clustering the reads (*e.g.* re-assign errors to the parental sequences it arose from) and merging the forward and reverse

read[5]. The first step was to trim away bases at the end of the reads that had low quality, *i.e* a phred score bellow 30, based on the MultiQC output as well as the quality plots within the DADA2 pipeline. For the forward reads Primer combination 1 and 2 had high enough quality and hence did not need any further trimming, but for primer combination 3 the reads were cut to 200 bp. The reverse reads were cut to 150 bp (primer combination 1), 131 bp (primer combination 2, both gDNA and cDNA), 120 bp (primer combination 3) respectively. Next step was to identify and remove any sequences that still remained in the data set which had an overall too low quality. For this DADA2 uses maximum number of expected errors instead of average quality score. The expected errors are calculated as the mean number of errors that would be observed in a large pool of sequences given that the error rate at each position is based on the quality score and that the errors at different positions are independent of each other[6]. Using maximum number of expected errors is an alternative to using average quality score in determining if a given read have high enough quality or if it should be removed from further analysis. The maximum number of error was here set to 2 in the forward read and 3 in the reverse read. To check that these were good thresholds the quality was checked with FastQC and MultiQC (most sequences had a quality of 30 or above). DADA2 then preforms a clustering step, in order to re-assign errors to the parental sequences that they arose from. Briefly describe, DADA2 starts with the most abundant sequences and then cluster putative errors around this sequence. Error rates are estimated for the data and used, along with the abundance of the sequences, to calculate the probability that the putative error have arisen from the parental sequences. Based on this the sequences are either merged or the putative error is defined as real sequences and moved to a new cluster. This is repeated until no sequences are left in the data set that have a p-value that corresponds to having high probability of being a real sequence. After the clustering the forward and the reverse reads were merged. Finally, chimeric sequences were identified and removed from the data set.

Since DADA2 was not originally designed to filter MHC data, we double checked the chimera results by running the program once with and once without the chimera checking step and then comparing the results. Most sequences identified as chimeras had low frequencies and since chimeric sequences should occur at lower frequency these were discarded. But four sequences in the gDNA data (three with primer combination 1 and one with primer combination 2) had high frequencies and these were checked manually. These sequences could not be explained as chimeras (they had unique bases) and where thus added to the final data set.

After this, the data was further filtered, still separate for each primer combination and DNA type. For gDNA, first any sequences with different lengths than expected, and not varying by three bases, were deleted. For primer combination 2, there seemed to be a general error where an extra base was kept at the start of the sequence, this happened in 15% of the sequences, this extra base pair was removed and then the sequences were kept. Next, a minimum per amplicon frequency threshold was determined and any sequences having a frequency below this threshold was deleted form the data set. Since errors should occur in low frequencies this threshold was determined by identifying a clear jump in frequency. The threshold was set to 1% for primer combination 1 and 3% for primer combination 2. To check that this was a good threshold, the replicates were compared and all matched 100%. All sequences remining after this were considered true alleles. For the cDNA, the filtering was done in a different way since here we wanted to keep alleles that had a low per amplicon frequency. First sequences with the wrong length were removed (again primer combination 2 had sequences with one extra base pair in the beginning). The cDNA data was then compared to the gDNA data (since all expressed alleles should also be found in the gDNA data) and all alleles that had previously been found within an individual in the gDNA were considered to be real expressed

alleles. The next steps were to determine if the sequences that had not been amplified in the gDNA should be considered real or artefacts. A threshold was set based on the average read depth for each primer combination and corresponded to that each allele should have at least 100 reads (0.25% for primer combination 2 and 0.3% for primer combination 3). All sequences remaining after these filtering steps were considered real expressed alleles.

References

1. Drews, A., Strandh, M., Råberg, L. & Westerdahl, H. Expression and phylogenetic analyses reveal paralogous lineages of putatively classical and non-classical MHC-I genes in three sparrow species (Passer). *BMC Evol. Biol.* **17**, 152; 10.1186/s12862-017-0970-7 (2017).
2. Martin, M. Cutadapt removes adapter sequences from high-throughput sequencing reads. *EMBnet.journal* **17**, 10; 10.14806/ej.17.1.200 (2011).
3. Andrews, S. FastQC: a quality control tool for high throughput sequence data. (2010). Available at: https://www.bioinformatics.babraham.ac.uk/projects/fastqc/.
4. Ewels, P., Magnusson, M., Lundin, S. & Käller, M. MultiQC: Summarize analysis results for multiple tools and samples in a single report. *Bioinformatics* **32**, 3047–3048 (2016).
5. Callahan, B. J. *et al.* DADA2: High-resolution sample inference from Illumina amplicon data. *Nat. Methods* **13**, 581–583 (2016).
6. Edgar, R. C. & Flyvbjerg, H. Error filtering, pair assembly and error correction for next-generation sequencing reads. *Bioinformatics* **31**, 3476–3482 (2015).
